# Functional MRI reveals subcortical auditory push–pull interactions requiring intercollicular integrity

**DOI:** 10.1162/IMAG.a.155

**Published:** 2025-09-22

**Authors:** Frederico Severo, Mafalda Valente, Noam Shemesh

**Affiliations:** Champalimaud Research, Champalimaud Foundation, Lisbon, Portugal

**Keywords:** fMRI, negative BOLD, subcortical auditory processing, auditory push–pull, inferior colliculus

## Abstract

The role of subcortical structures in binaural integration is of great interest for auditory processing. The inferior colliculus (IC) is the main auditory midbrain center where ascending and descending auditory projections converge, which was suggested to encode auditory information via a push–pull mechanism (a coordinated antagonistic neural mechanism for adaptive response control) between the two ICs. However, the origin of this push–pull mechanism in the brain and how it interacts with other upstream/downstream subcortical areas are still a matter of great debate. Here, we harness functional MRI (fMRI) in combination with IC lesions in the rat to dissect the push–pull interaction from a pathway-wide perspective. We find evidence for the push–pull mechanism in IC through opposing negative/positive fMRI signals in the ipsilateral/contralateral ICs upon monaural stimulation. By unilaterally lesioning the corresponding contralateral IC, we demonstrate the necessity of collicular integrity and intercollicular interactions for the push–pull interaction. Using binaural stimulation and IC lesions, we show that the push–pull interaction is exerted also in binaural processing. Finally, we demonstrate that, at least at the population level revealed by fMRI, the main push–pull interactions occur first at the IC level, and not earlier, and that the outcome of the push–pull “calculation” is relayed downstream to the medial geniculate body (MGB). This dissection of the push–pull interaction sheds light into subcortical auditory function.

## 1 Introduction

Sound processing plays a vital role in guiding behavior and interaction with the environment, particularly in navigation, spatial awareness, and the localization of sound sources (Dent et al., 2018; Malmierca, 2015; O’Shaughnessy, 1957). It allows organisms to detect, identify, and respond to dynamic acoustic cues in real time, supporting tasks such as orienting toward or away from stimuli, communicating with conspecifics, and forming an accurate perceptual map of their surroundings. Several subcortical brain structures (Supplementary Fig. S1) are directly involved in monaural/binaural integration and processing, but the inferior colliculus (IC) is thought to play an especially critical role. The IC is the principal source of input to the auditory thalamus, receiving excitatory, inhibitory, and modulatory inputs (Ayala et al., 2016; Ito & Oliver, 2012) from the entire auditory pathway and integrating the parallel pathways emerging from the cochlear nucleus (CN) (Malmierca, 2006). Evidence indicates that the left and right ICs function together, as the largest afferent source to each IC has been suggested to be the contralateral IC, through excitation, inhibition, or a combination of both (Caicedo & Herbert, 1993; Kuwabara & Zook, 2000; Saldaña & Merchán, 1992). A “push-pull”-like mechanism (a functional dynamic in which two distinct brain regions exhibit opposing neural responses to the same stimulus or task) (Xiong et al., 2013) has been proposed for IC function, with contralateral IC (cIC) excitation balanced by ipsilateral IC (iIC) inhibition, as well as evidence suggesting an intercollicular neural pathway modulating neural responses, both within the IC itself (Liu et al., 2022; Orton & Rees, 2014) and MGB (Mellott et al., 2014). Interestingly, early evidence of this “push-pull” interaction can be traced back decades (Hind et al., 1963), yet most of its mechanisms, dynamics, and relationships with activity in other parts of the pathway remain unclear. Several studies have identified the superior olivary complex (SOC) as potentially the first brain structure involved in this mechanism (Casseday et al., 2002; Kavanagh & Kelly, 1992; Moore & Caspary, 1983), while others linked it to inhibitory input from the medial geniculate body (MGB) onto SOC neurons (Cant & Casseday, 1986; Casseday et al., 2002; Pollak, 2012) or to the influence of the contralateral inferior colliculus (IC) modulating ipsilateral neuronal responses during binaural hearing (Liu et al., 2022). Traditional methods used to investigate these push–pull interactions such as electrophysiology are invasive, rendering simultaneous studies of multiple structures, particularly of subcortical areas, challenging. However, behavioral studies (Brunton et al., 2013; Jaramillo & Zador, 2014; Pardo-Vazquez et al., 2019), while providing a general perspective in auditory processing and behavioral phenotypes, do not provide insights into activity in these subcortical areas.

Functional magnetic resonance imaging (fMRI) enables the investigation of global brain function via the blood oxygenation level-dependent (BOLD) coupling mechanism (Ogawa et al., 1990), which can be considered a surrogate reporter of underlying neural activity (Dubois et al., 2015; Gil, Valente, & Shemesh, 2024; Goense & Logothetis, 2008; Siero et al., 2014). Despite the possibility of whole brain imaging, fMRI studies related to sound localization in humans have overwhelmingly focused on the role of cortical structures, likely because task-based human auditory processing is predominantly cortical in nature (Poeppel et al., 2012; Steinschneider et al., 2013). In contrast, the rat subcortex comprises a much larger brain fraction relative to humans, making it a suitable animal model for the study of subcortical structures (Xu et al., 2022). fMRI has been previously used to characterize the intact auditory pathway in mice (Blazquez Freches et al., 2018) and rats, mapping features such as tonotopy (Cheung et al., 2012), sound pressure level encoding (Zhang et al., 2013), or laterality (Lau et al., 2013). In these studies, strong positive BOLD-fMRI responses were observed along the different structures of the auditory pathway, mostly contralaterally to the presented sound. No ipsilateral responses (barring CN) or evidence of an auditory push–pull mechanism has been previously reported using BOLD fMRI in rodents, despite the electrophysiological evidence suggesting reciprocal patterns of activity, such as excitation in one region coupled with inhibition in another. Indeed, push–pull interactions can manifest in various physiological forms, including excitatory versus inhibitory neural signaling (Liu et al., 2022; Xiong et al., 2013), increased versus decreased neuronal firing rates (Gil, Valente, & Shemesh, 2024), or contrasting patterns of blood-oxygen-level-dependent (BOLD) signals, such as positive BOLD responses in one area coinciding with negative BOLD responses in another (Gil, Valente, Fernandes, et al., 2024). Recently, high-field rodent MRI systems and cryogenic probes were shown to sufficiently enhance sensitivity toward detecting negative BOLD signals upon population-level silencing, with electrophysiological data showing strong deactivation of the areas tied to negative BOLD responses during rapid visual stimulation (Gil, Valente, Fernandes, et al., 2024; Gil, Valente, & Shemesh, 2024), suggesting a potential avenue for exploring similar patterns in other systems.

**Fig. 1.**
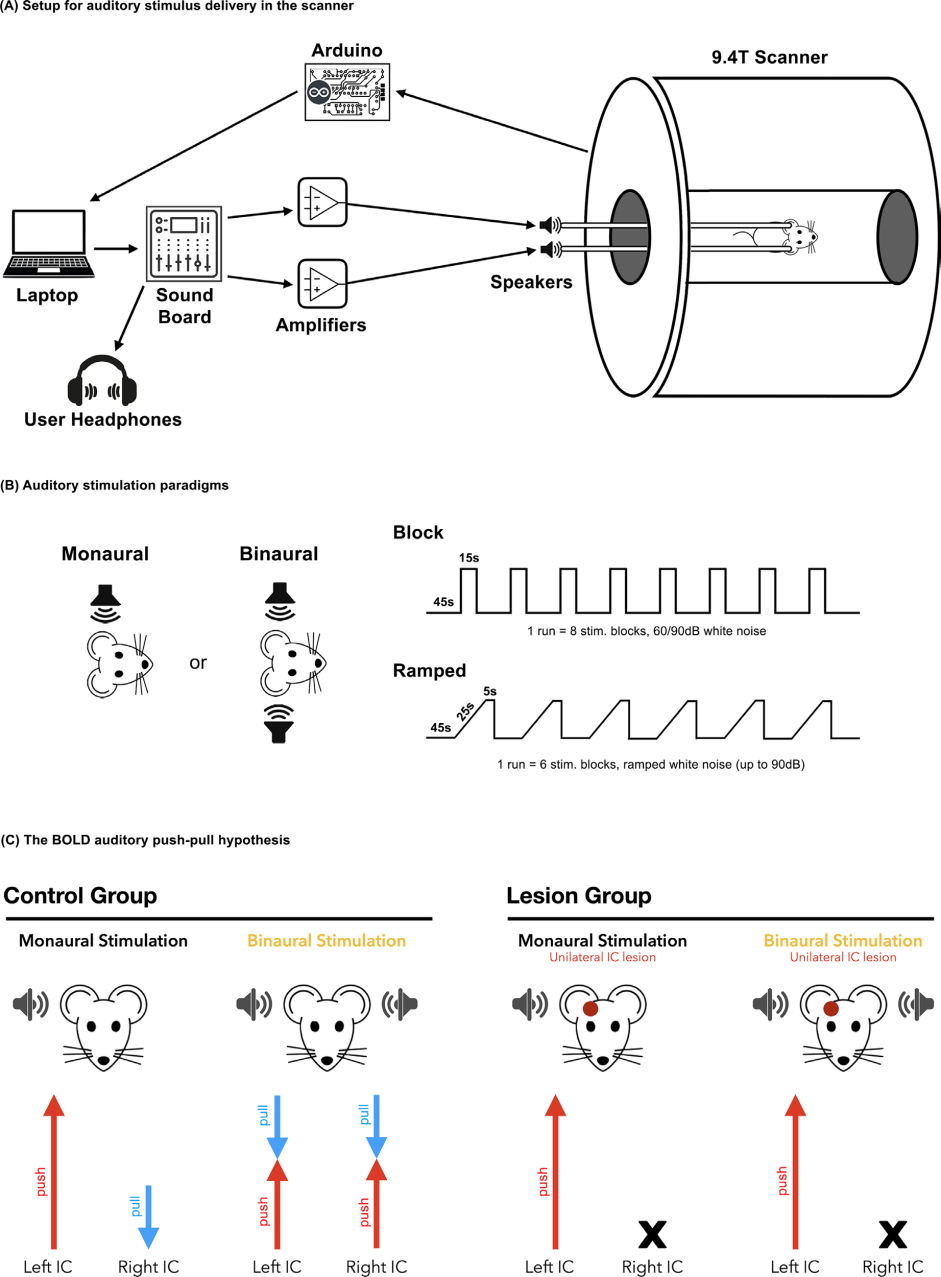
The auditory pathway and experimental design. (A) Schematic for the auditory stimulus delivery system in the scanner, depicting the auxiliary computer which generates the sounds, sound board, amplifiers, speakers docked to the main tubes for sound delivery, and circuit board for TTL control. (B) Auditory stimulation paradigms (block and ramped). Broadband white noise (5–45 kHz) was used in every experiment, regardless of the paradigm. (C) The BOLD auditory push–pull hypothesis, showing the different dynamics upon monaural/binaural responses in control and IC lesioned groups. Red arrows represent positive BOLD, while blue arrows represent negative BOLD signals. X represents lack of activation in the lesioned area.

Leveraging the experimental flexibility of rodent models, we aimed to investigate the detectability of push–pull dynamics within the inferior colliculus and their broader impact on subcortical auditory processing. To this end, we employed auditory-evoked fMRI combined with a targeted lesion model, allowing us to examine how unilateral disruption of the IC alters mono/binaural patterns of activity across the auditory pathway. We demonstrate robust, positive BOLD responses in contralateral auditory structures and consistent negative BOLD responses in the ipsilateral IC, supporting the existence of push–pull dynamics in the rodent auditory midbrain. Our lesion model further demonstrates that intact intercollicular pathways are essential not only for the generation of negative BOLD responses in the inferior colliculus but also for maintaining the normal dynamics between monaural and binaural auditory processing. The disruption of these pathways through unilateral lesions abolishes the typical push–pull pattern, highlighting the critical role of cross-hemispheric communication in preserving the balance and asymmetry of auditory responses (Fig. 1C).

## 2 Materials and Methods

In this study, we use functional MRI to investigate the subcortical auditory pathway in rats, with a particular focus on the auditory push–pull mechanism. Our approach begins with monaural sound stimulation, which allows us to map the activation patterns along the ascending auditory pathway and establish a baseline of how unilateral input is processed. Building on this, we explore a novel finding: a negative BOLD response in the ipsilateral inferior colliculus. To better understand the nature and significance of this signal, we systematically manipulate the acoustic stimulus itself and introduce a lesion model to perturb the system and observe resulting changes in neural dynamics. Finally, we examine how binaural stimulation alters these patterns, allowing us to assess how bilateral input modulates the push–pull interaction across the pathway.

### 2.1 Animal model

Adult Long Evans, weighing between ~200–350 g and aged 8 to 16 weeks old, housed under ad libitum food and water, under normal 12 h/12 h light/dark cycle, were used for the auditory fMRI experiments. In this study, a total of N = 43 rats were used (27 females). Healthy (control) animals consisted of N = 24 (negative BOLD (N = 6), ramped (N = 6), monaural vs binaural (N = 6), isoflurane (N = 6)). Lesioned animals (a separate cohort) consisted of N = 19 (lesion model (N = 6), monaural vs binaural (N = 6), sham lesion (N = 2), VC lesion (N = 2), histology (N = 3)). This study included both male and female animals, without applying sex-based selection criteria, as sex was not expected to significantly influence the outcomes of the experiments. All animals were naïve to auditory stimulation, meaning they had no prior exposure to the white noise stimuli used in our paradigms, nor to the scanner acoustic environment, ensuring that responses were not shaped by previous auditory experience or habituation. Auditory maturity in tonotopy, threshold and timing, characterized by adult-like auditory processing capabilities, is typically achieved by P20 to P25 (Geal-Dor et al., 1993). The developmental sharpening of intracortical inhibition effects occurs later than excitatory input, being complete by P45 (~6 weeks) (Froemke & Jones, 2011). All animal care and experimental procedures were carried out according to the European Directive 2010/63 and pre-approved by the competent authorities, namely, the Champalimaud Animal Welfare Body and the Portuguese Direcção-Geral de Alimentação e Veterinária (DGAV).

### 2.2 Setup for delivery of auditory stimulus in the scanner

The setup depicted in the Figure 1A schematic was designed to deliver precise auditory stimuli inside the magnet. A 2-channel soundboard (YAMAHA AG-03, Shizuoka, Japan) with a dynamic range of 24 bits and 2.451 VRMS output before clipping was used to interface the white noise generated in Matlab and 2 in-house designed voltage amplifiers capable of 3x amplification up to 24 peak-to-peak voltage (Vpp). To deliver the sound, a piezoelectric speaker (L010 KEMO, Leher Landstr Geestland, Germany) capable of producing ultrasonic sounds at high output levels (~100 dB) with a relatively flat frequency response up to 75 kHz (Cheung et al., 2012) was placed outside of the scanner bore, both due to size restrictions and metallic components. To guide the sound waves into the rat’s ears and allow for optimal placement of the rat in the cryocoil, specialized tubing was required. The interface between the speaker and rat was accomplished by a polyethylene connector (Geolia, Lezennes, France) and a 90 cm length, 4 mm wide polyethylene tube that connected to a custom-made curved earpiece that inserts into the rat’s ear. The earpieces were kept in place by a screw mechanism connected to the cryocoil bed, as well as medical tape placed over the animal’s ears. For all experiments, the piezoelectric speakers were calibrated for an approximately flat response to white noise with a custom-made Matlab script, using a free-field reference microphone with a Type 2670 preamplifier (Brüel & Kjær, Nærum, Denmark), capable of a flat frequency response from 4 Hz to 100 kHz. The generated white noise (Supplementary Fig. S2D) was measured at the distal end of the polyethylene tube and earpiece assembly, outside the scanner environment. While the acoustic interface (tubing, earpiece, and coupling mechanisms) did introduce some alterations to the overall sound quality, this effect was anticipated and accounted for during the calibration process. Calibration was performed with the full auditory setup in place to ensure that the delivered sound pressure levels at the ear closely matched the intended stimuli, thereby preserving the integrity and reproducibility of the auditory stimulation across experiments.

As the sound is presented to the animal through dedicated earpieces pointed directly at the ear canal, the acoustic shadow of the ears, head, and torso of the rat, present in a more natural setting when the sound source is presented at a distance from the animal, is bypassed. Thus, a purely diotic stimulus is presented, with simultaneous stimulation of both ears with the same sound, unobstructed and mostly unaltered by interactions with the rat’s body. The time profiles of sound pressure waves were also measured to ensure sounds were presented to both ears at the same time in order for interaural time differences to not significantly affect the results of this study.

Two speakers were used to enable independent delivery of monaural and binaural auditory stimuli, allowing for flexible configurations including left-only, right-only, and combined (left + right) stimulation. A batch of 20+ speakers was initially tested and paired based on matched frequency response characteristics to ensure consistency between the left and right channels. Although individual variation across the batch was minimal within the relevant stimulation frequency range, careful pairing was done to optimize acoustic fidelity. Synchronization between the two speakers was verified using an oscilloscope, with measurements taken post-mixer and pre-amplification to ensure precise timing alignment of audio signals. It was assumed that the remaining components of the auditory delivery setup, including the amplifiers and polyethylene tubing (which was cut to equal lengths for both sides), did not introduce any measurable latency differences between channels. To maintain the reliability and accuracy of the system, frequency response, amplitude balance, and synchronization were periodically re-tested throughout the course of the experiments.

Scanner interfacing was achieved using an Arduino microcontroller (ARDUINO, Officine Arduino/Fablab Torino, Via Egeo 16, 10134 Turin, Italy), which functioned as a trigger detection device. Trigger signals were generated by embedding specific trigger lines into the MRI pulse sequences, programmed to emit a signal at the precise moment when an auditory cue was scheduled to occur, ensuring accurate synchronization between the scanner and the auditory stimulus delivery system. Pulse sequence sound spectra were also recorded, at 30 cm from the magnet bore (Supplementary Fig. S2A–C).

### 2.3 Auditory paradigms

For auditory stimulation, broadband white noise 5–45 kHz was presented into the animal’s ears, using our specialized setup (Fig. 1A). White noise was chosen for being a simple, “meaningless” stimulus to rodents (Soga et al., 2018), devoid of tonotopy preference, but still salient enough to produce a reliable response in subcortical structures (Cheung et al., 2012).

Two stimulation paradigms, both in the form of a standard block design, were used throughout this study (Fig. 1B): block and ramped. The on/off white noise block paradigm was used because it is the standard approach in auditory research, allowing for more direct comparisons with other studies, and has been shown to produce consistent responses throughout the auditory pathway. It consisted of 8 blocks of 15 sec stimulation and 45 sec rest (starting with a rest period), for a total scan time (per run) of 8 min 45 sec. Auditory stimulation was randomized for each run between monaural (left or right) and binaural stimulation at 90 dB (60 dB for control experiments where specified) for animals in the control and lesioned group.

Additionally, we employed an amplitude-modulated auditory stimulus to investigate how the IC in each hemisphere, contralateral and ipsilateral to the stimulated ear, differed in its temporal response dynamics and sensitivity to changes in sound amplitude. This approach allowed us to probe not only the overall activation patterns but also how each side of the IC tracks the temporal envelope of the stimulus. This modulated ramped paradigm consisted of 6 blocks of 30 sec stimulation and 45 sec rest (starting with a rest period), for a total scan time (per run) of 8 min and 15 sec. The 30 sec stimulation was composed of 2 parts, a 25 sec amplitude ramped noise that goes from 0 to 90 dB, followed by a 5 sec plateau at 90 dB. The ramps are themselves modulated, having different envelopes (Supplementary Fig. S6), termed “Late Rise,” “Intermediate,” and “Early Rise” based on their characteristics. For each run, auditory stimulation was randomized between the three possible ramp envelopes, and left- or right-sided monaural stimulation.

### 2.4 Surgery for lesions

Before surgery, each animal was scanned and a whole brain, high-resolution anatomical T_2_-weighted set of images was acquired (c.f. Subsection MRI for specific details). These images were then aligned and superimposed with an atlas reference for stereotaxic coordinates (Paxinos & Watson, 2009) for surgery planning (it is important to note that the Paxinos atlas is based on adult male Wistar rats weighing approximately 300 g, whereas our experimental cohort included animals of varying weights and both sexes). These anatomical variations were taken into consideration during registration to minimize discrepancies in targeting accuracy. The lesion side (left or right) was alternated between animals. For surgery, the animal was first deeply anesthetized with 5% isoflurane (Vetflurane, Virbac, France) for 2 min and then moved to a stereotaxic setup (KOPF Model 1900, David Kopf Instruments, CA, USA). During surgery, the animal was kept under 3–2.5% isoflurane, and its temperature was kept within physiological parameters with the aid of an active charcoal heating pad (Little Hotties Hand Warmers, Implus, NC, USA).

The stereotaxic coordinates for the lesions were determined for each individual rat in order to maximize the likelihood of a successful lesion and thus coordinates varied across animals. Injections in the IC were performed in 3 AP locations (-7.8 ± 0.2 AP; -8.6 ± 0.4 AP; -9.2 ± 0.15 AP), each with 3 ML injection points (for the first AP coordinate: 1.4 ± 0.3 ML, 1.6 ± 0.5 ML, and 2.3 ± 0.7 ML; for the second AP coordinate: 1.2 ± 0.1 ML, 1.9 ± 0.1 ML, 2.6 ± 0.1 ML; and for the third AP coordinate: 1.1 ± 0.1, 1.8 ± 0.1, 2.2 ± 0.4) and at one or two depths, ranging between -1 ± 0.6 DV and -3.9 ± 0.3 DV. These coordinates were measured in relation to Bregma and the dorsoventral coordinates had their origin in the surface of the brain. For VC targeting, we used the coordinates from (Gil, Valente, & Shemesh, 2024). For the neural lesion we used ibotenic acid (Pai et al., 2011) injected with a NanoJet II (Drummond Scientific Company, Bromhall, USA) through pulled glass pipettes, to avoid mechanical damage to the tissue, we used extremely thin (OD = 10–25 μm) pipettes (Augustinaite & Kuhn, 2020; Gonzalez-Perez et al., 2010; Pai et al., 2011). Ibotenic acid is an excitotoxin that damages cells by causing a large influx of calcium into the cell, creating an excessive release of glutamate, and activating excitatory plasma membrane receptors (Neves et al., 2023). It mainly targets excitatory neurons (McQueen, 2010; Raghavendra et al., 2013) which leads to a lesioned area where a diffuse border of sparser cell density or even with no intact neurons can been identified (Pai et al., 2011). In each defined coordinate, the solution was injected in pulses of 32 nL at a rate of 23 nL per second, the total injected volume was dependent on the IC size for each specific animal with a maximum total volume injected of 608 nL. For the deeper injection coordinate, the needle was kept in place for 5 min following infusion and then pulled up to the more superficial coordinate, where it remained for 10 min after infusion to avoid propagation of the acid to more superficial structures. After the injections, the craniotomy was covered with a silicone elastomer sealant (kwik-cast™, World Precision Instruments, USA) layer. Post-surgery, the animal was injected, subcutaneously, with 5 mg/Kg body weight of carprofen (Rimadyl ®️, Zoetis, USA) and the incision sutured. Our lesion model is designed to reflect an acute time point of IC inactivation. Lesioned animals were single housed to reduce stress and interactions with external sound sources. MR scanning then took place ~24 h post-surgery. At this time point, inflammation at both the injection site and the cranial surface can still be pronounced, with subdermal fluid accumulation requiring postoperative drainage in some cases. Sham (saline in IC) and control lesions in the visual cortex (VC) were also performed to account for effects of the surgery itself (Supplementary Fig. S7), and global effects of the ibotenic acid. The same protocols were followed. Location and extent of the lesions were confirmed following the same protocol as the anatomical T_2_ weighted prescans. Lesion progression was consistent with previous lesion studies conducted in our laboratory, in which animals received targeted lesions in various brain regions, including auditory cortex, superior colliculus, and visual cortex (Gil, Valente, Fernandes, et al., 2024; Gil, Valente, & Shemesh, 2024).

### 2.5 Histology

A separate cohort of animals (n = 3) were perfused transcardially, ~24 h after the lesion was performed, first using 250 to 350 ml of PBS 1x, followed by similar volume of 4% PFA. First with a PBS 1x solution, followed by 4% PFA. The brain was extracted and kept in 4% PFA for approximately 12 h. After this, the brain was placed in a 30% sucrose solution for a minimum of 4 days, after which the tissue was embedded in O.C.T. compound (FSC 22, Leica Biosystems, Nussloch, Germany) and frozen to be sliced on a cryostat and sliced on a cryostat (Leica CM3050S, Leica Biosystems, Nussloch, Germany). After sectioning, the brain slices were stained with cresyl violet, mounted with mowiol mounting medium and imaged in a Zeiss AXIO Imager M2 microscope (Carl Zeiss Microscopy, Thornwood, NY, USA).

### 2.6 Animal preparation for fMRI

Rats were anesthetized briefly with 5% isoflurane (VIRBAC, Carros Cedex, France) maintained by a vaporizer (VETEQUIP, Livermore, CA, USA) in a custom-built box. The isoflurane concentration was reduced to ~ 4% after ~ 2 min, and the animals were quickly moved to the cryocoil animal bed and stabilized with a nose cone and a bite bar. Around 5 min after induction, a bolus of medetomidine solution 1:10 dilution of 1 mg/ml medetomidine solution in saline (VETPHARMA ANIMAL HEALTH S.L., Barcelona, Spain) was administered by subcutaneous injection (bolus = 0.05 mg/kg, (GenieTouch, Kent Scientific, Torrington, CT, USA)). The earpieces were then carefully placed and angled pointing at the rat’s ear canal, and kept in place by the previously described screw mechanism, effectively having the earpieces double as makeshift ear bars. To further keep the earpieces from moving and reduce external sound, the animals’ ears were filled with vaseline-doused cotton pieces (after the insertion of the earpiece), and taped down to the screw. After assembly, the bed was then inserted to the scanner. After 15 min (10 min after the bolus injection), a constant subcutaneous infusion of medetomidine was started, 0.1 mg/kg/h, delivered via a syringe pump. Isoflurane dosage was progressively reduced to 0% in 15 min and kept at 0% throughout the remainder of the MRI session. To achieve efficient isoflurane washout, acquisitions were always started between 50 and 60 min after bolus injection. During the entire time course of the experiments, animals breathed oxygen-enriched medical air composed of 71% nitrogen, 28% oxygen, and the remaining 1% comprising mostly argon, carbon dioxide, and helium. Respiratory rate and temperature were monitored using a respiration pillow sensor (SA Instruments Inc., Stony Brook, NY, USA) and an optic fiber rectal temperature probe (SA Instruments Inc., Stony Brook, NY, USA). Each experiment lasted about 3.5 h. In the end of the experiment, a 5 mg/ml solution of atipamezole hydrochloride (VETPHARMA ANIMAL HEALTH, S.L., Barcelona, Spain) diluted 1:10 in saline was injected subcutaneously with the same volume as for the medetomidine bolus to revert the sedation.

### 2.7 MRI

All data in this study were acquired using a 9.4T Bruker BioSpin MRI scanner (Bruker, Karlsruhe, Germany), equipped with an AVANCE III HD console with a gradient unit capable of producing pulsed field gradients of up to 660 mT/m isotropically with a 120 µs rise time. Radiofrequency transmission was achieved using an 86 mm quadrature coil, while a 4-element array cryoprobe (Baltes et al., 2009) (Bruker, Fallanden, Switzerland) was used for reception. The software running on this scanner was ParaVision^®^ 6.0.1.

### 2.8 Positioning and pre-scans

Following localizer scans ensuring optimal positioning of the animal and routine adjustments for center frequency, RF calibration, acquisition of B_0_ maps, and automatic shimming using the internal MAPSHIM routine, a high-resolution anatomical T_2_-weighted rapid acquisition was performed with refocused echoes (RARE) sequence (TR/TE = 1000/13.3 ms, RARE factor = 5, FOV = 20 × 16 mm^2^, in-plane resolution = 80 × 80 μm^2^, slice thickness = 500 μm, t_acq_ = 1 min 18 sec) was acquired for accurate referencing.

### 2.9 fMRI acquisitions

**Fig. 2.**
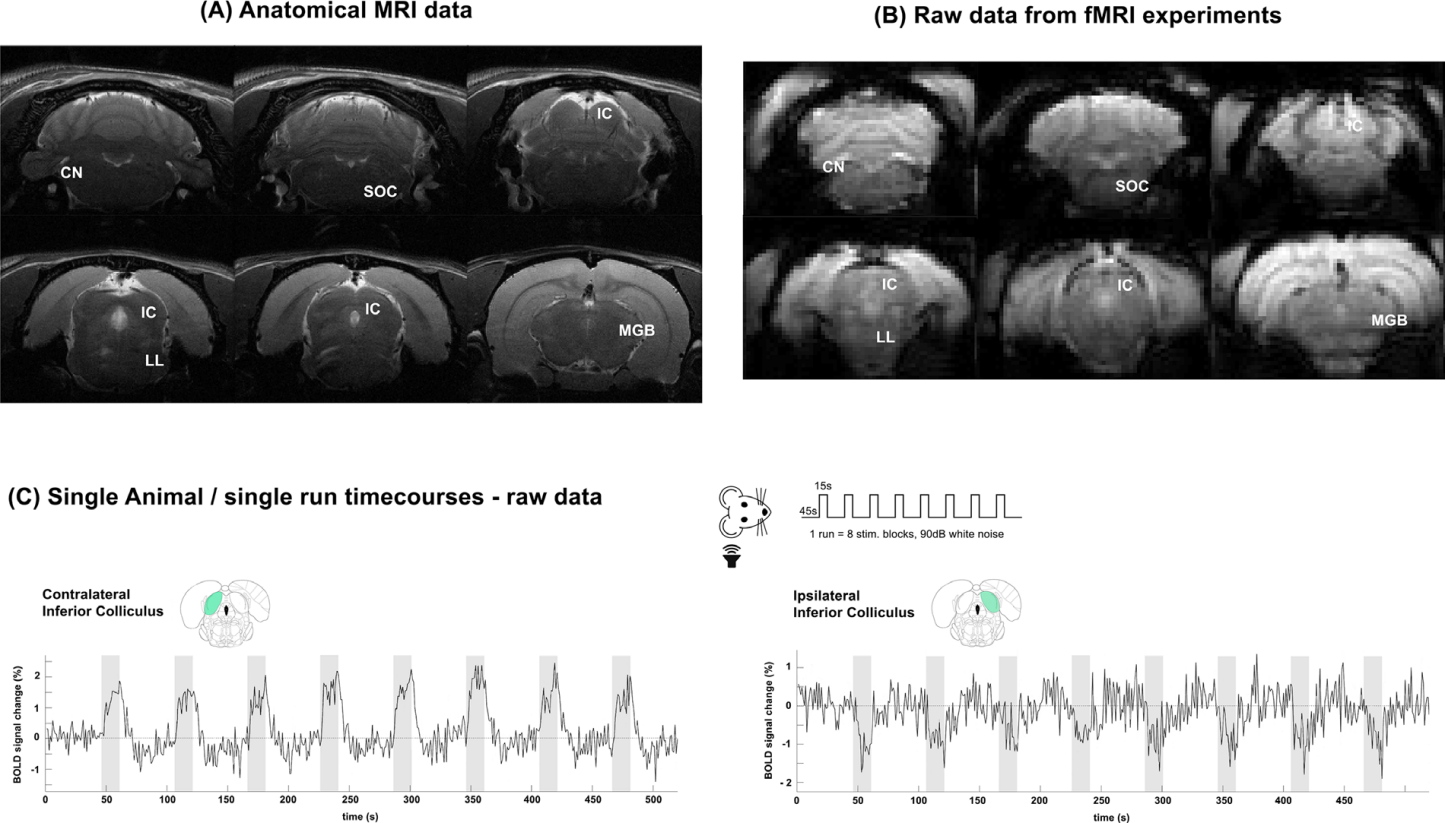
Anatomical and functional raw data. (A) Anatomical images of a representative animal, and the location of structures of interest in the subcortical auditory pathway. (B) Raw data from a representative fMRI experiment using a Gradient Echo EPI, presenting excellent SNR. Eight slices acquired, 6 slices shown after coregistration. (C) Time courses for ROIs placed in regions (contra/ipsilateral IC) along the pathway in a single rat and a single run reveal BOLD responses perceivable to the naked eye (a typical dataset is shown from one single representative rat). Green shade on brain atlas represents the structure of interest for each particular time course. Translucid gray bars indicate the stimulation periods.

**Fig. 3.**
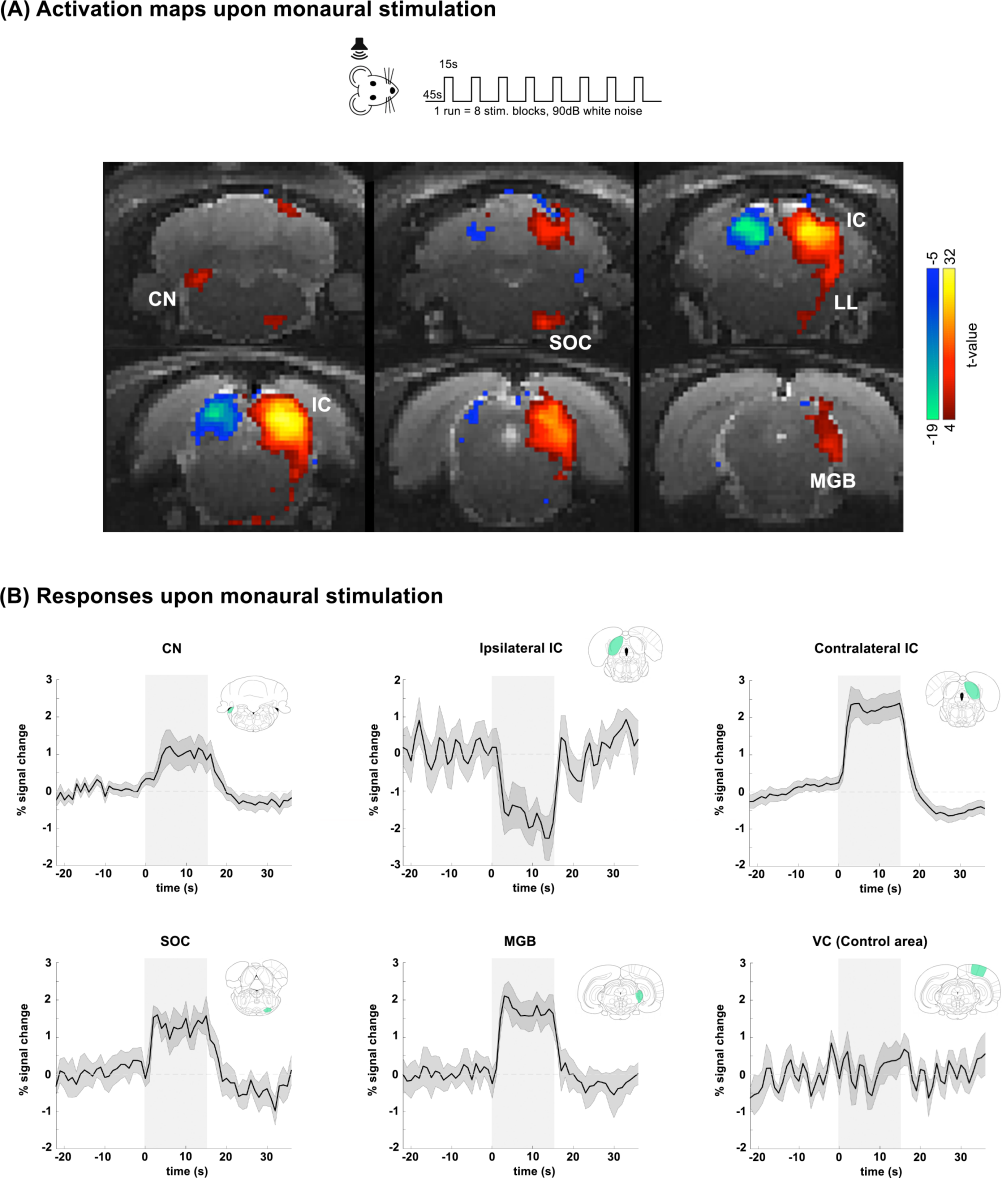
Monaural stimulation responses. (A) Activation maps of the subcortical auditory pathway upon monaural stimulation with white noise. N = 6 (8 slice acquisition). (B) Averaged time courses over cycles/animals, for ROIs placed in regions of the subcortical pathway upon monaural stimulation (and VC as a control area). Green shade on brain atlas represents the structure of interest for each particular time course. Translucid gray bars indicate the stimulation periods.

**Fig. 4.**
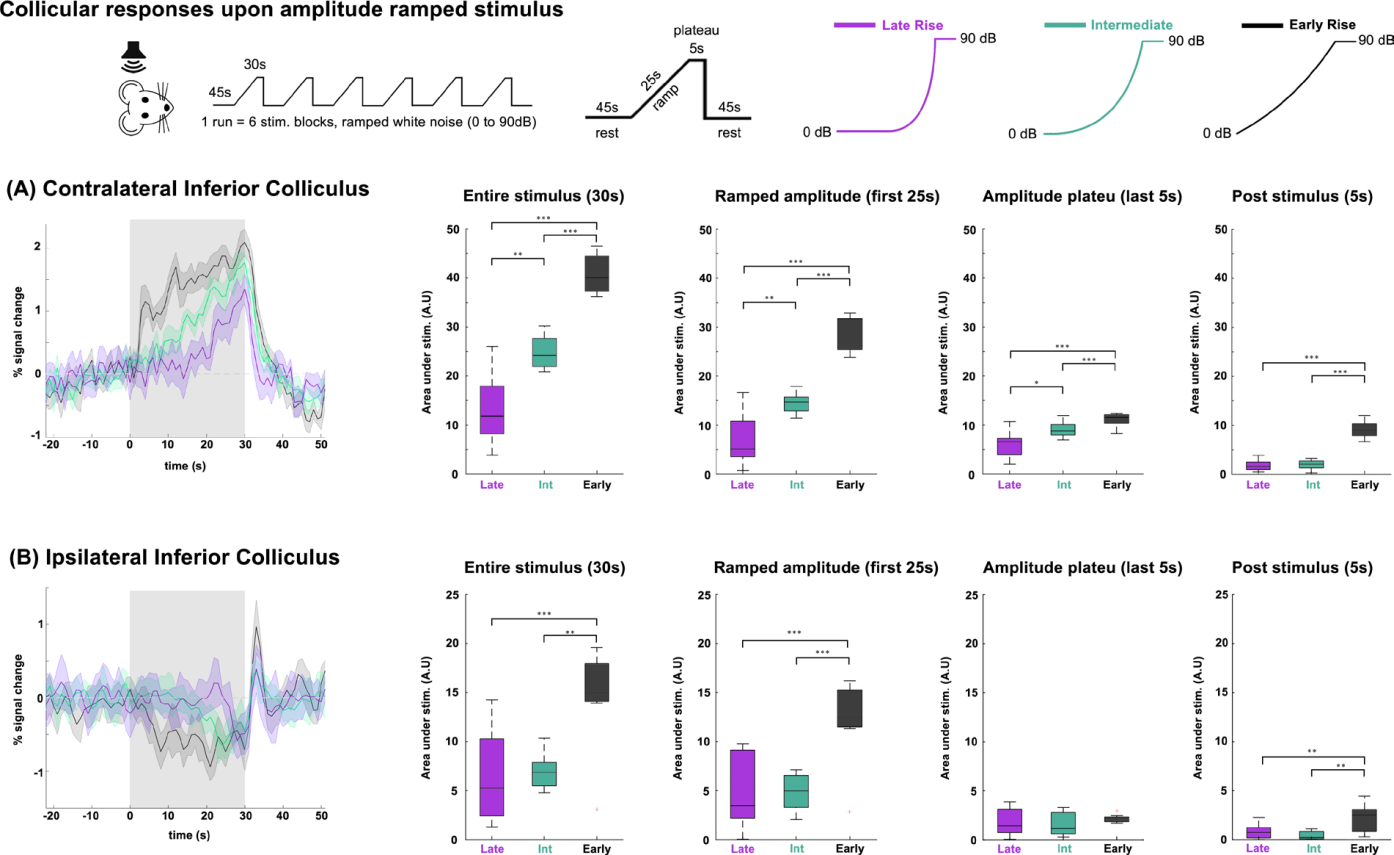
Collicular responses upon amplitude ramped stimulus. (A) Monaural ramped (Late Rise, Intermediate, and Early Rise) stimulus response on the contralateral inferior colliculus. The translucent gray bars indicate the stimulation periods. Comparison between groups used an ANOVA (Kruskal–Wallis) statistical test, ∗p ≤ 0.05; ∗∗p ≤ 0.01; ∗∗∗p ≤ 0.001. Green shade on brain atlas represents the structure of interest for each particular time course. Translucid gray bars indicate the stimulation periods. N = 6 (2 slice acquisition). (B) Monaural ramped stimulus response on the ipsilateral inferior colliculus. The translucent gray bars indicate the stimulation periods.

**Fig. 5.**
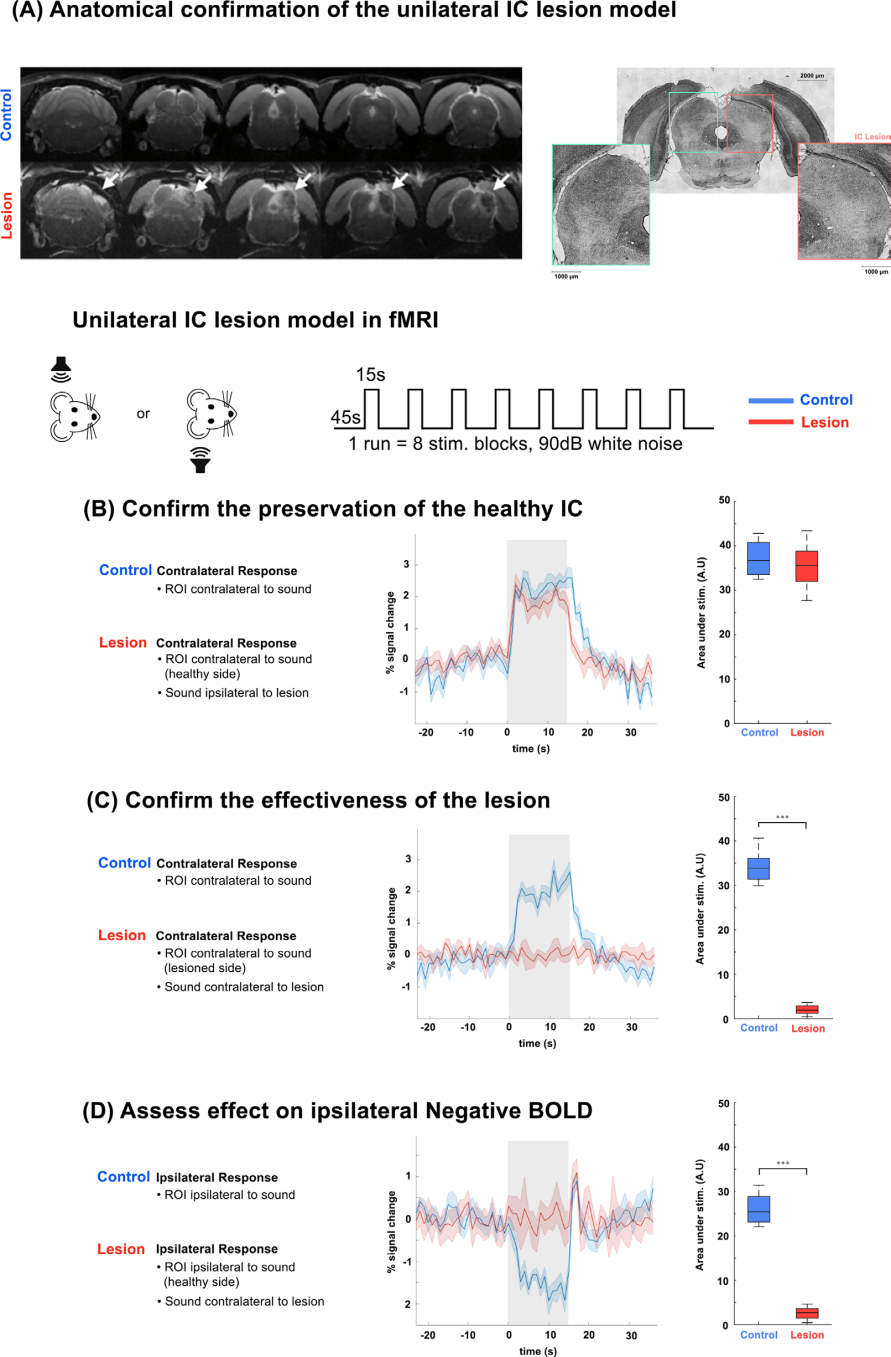
Unilateral IC lesion model in fMRI. (A) Anatomical comparison between a representative animal of the Control group and Lesion groups. White arrows point to the lesioned area. On the right, histology data show the healthy (green) and lesioned (red) colliculus, showing lowered neuronal density. Plots show time courses of BOLD responses two-sample parametric t-test, ∗p ≤ 0.05; ∗∗p ≤ 0.01; ∗∗∗p ≤ 0.001. Translucid gray bars indicate the stimulation periods. N = 6 (2 slice acquisition). (B) Confirming the preservation of healthy IC. Plots show time courses of BOLD contralateral responses to sound, with the ROI on the healthy IC. (C) Confirming the effectiveness of the lesion. Plots show time courses of BOLD contralateral responses to sound, with the ROI on the lesioned IC. (D) Assessing how the lesion modulates activity on the healthy side. Plots show time courses of BOLD ipsilateral responses to sound with the ROI on the healthy IC.

A gradient echo EPI (GE-EPI) sequence (TE/TR 14/1000 ms, PFT 1.5, FOV 20 x 13 mm^2^ in plane resolution 250 x 250 μm^2^, slice thickness 1 mm. The number of acquired slices varied with the particular experiment being run: 8 slices (Figs. 2 and 3; Supplementary Figs. S3, S5, S6, and S7), 2 slices (Figs. 4 and 5; Supplementary Figs. S7 and S8), and 1 slice (Supplementary Fig. S4), determined based on the anatomical extent of the primary area of interest and imaging constraints. In experiments where the focus was on a specific structure, such as the iIC, we deliberately limited the imaging field to a smaller, targeted region. This approach allowed us to achieve more precise shimming and improved magnetic field homogeneity over the area of interest, which was particularly important to highlight possible small differences between groups/conditions.

### 2.10 Data analysis

For the MRI data analysis, a general linear model (GLM) analysis was conducted along with a region of interest (ROI) time course analysis to investigate temporal dynamics of activation profiles. The use of GLM analysis in our workflow was intended primarily to facilitate visualization of activation patterns and provide an intuitive spatial overview of stimulus-driven responses. ROI time course analysis was used for quantitative assessment or statistical comparisons between conditions or regions. All data analysis was performed using Matlab (The Mathworks, Natick, MA, USA, v2016a and v2018b).

**Fig. 6.**
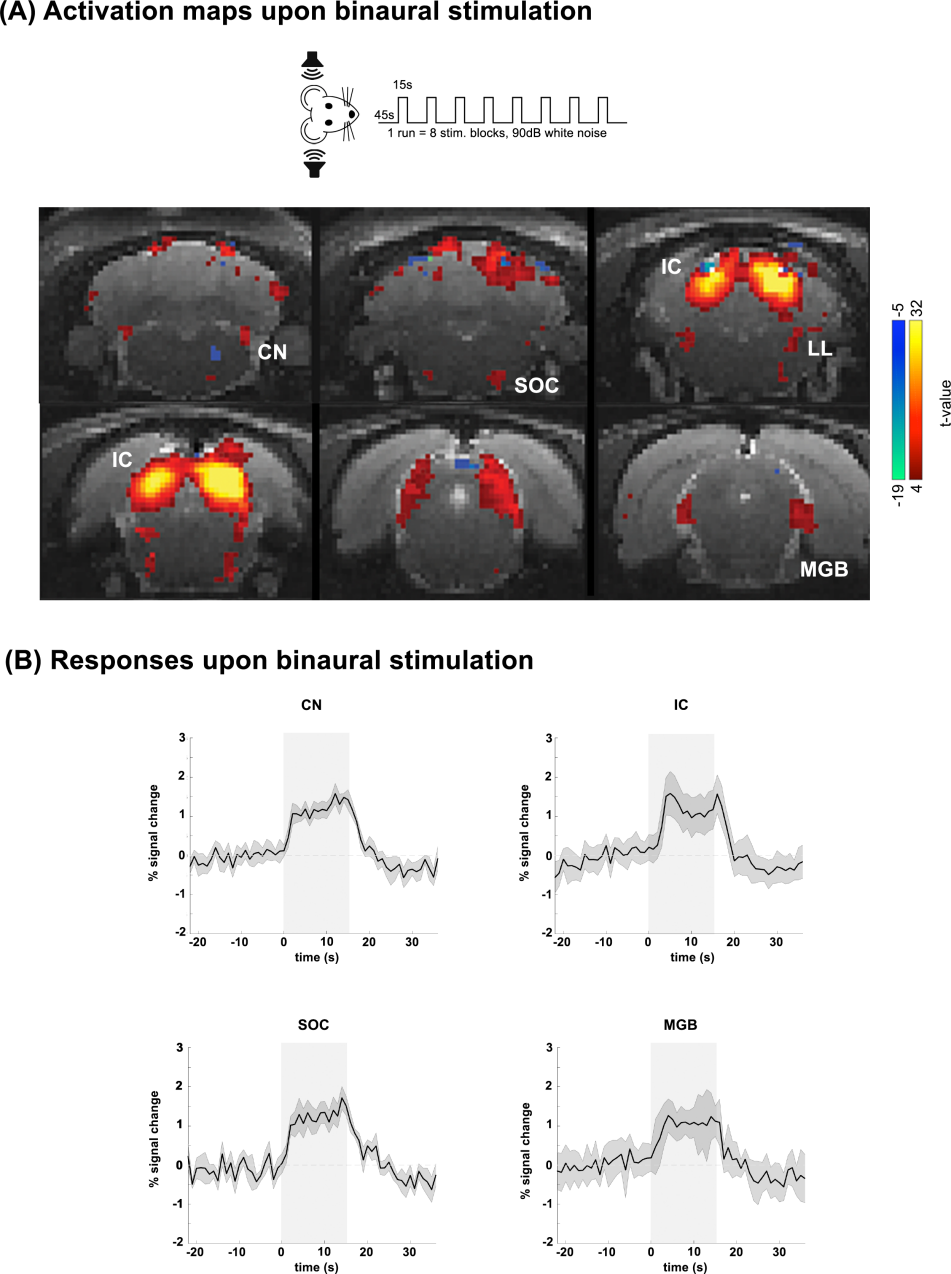
Binaural stimulation responses. (A) Activation maps of the auditory pathway upon binaural stimulation with white noise. N = 6 (8 slice acquisition). (B) Averaged time courses over cycles/animals (randomized subsample of all available acquisitions across both sides) for ROIs placed in structures of the pathway upon binaural stimulation. Green shade on brain atlas represents the structure of interest for each particular time course. Translucid gray bars indicate the stimulation periods.

GLM analysis was used to generate activation maps (statistical t-maps) for data visualization of monaural and binaural responses (Figs. 3 and 6). Preprocessing steps included manual outlier removal (<1% were identified as outliers, time points whose signal intensity was 3 times higher or lower than the standard deviation of the entire time course were replaced using spline interpolation taking the entire time course), slice timing correction (sinc interpolation) followed by head motion correction (using mutual information) and detrending, in order to remove low frequency trends. Data were then coregistered to the T_2_-weighted anatomical images, normalized to a reference animal (from each group) and smoothed using 3D Gaussian isotropic kernel with full-width half-maximum corresponding to 1 voxel (0.250 mm in plane, 0.500 mm in the slice direction). A fixed-effects group analysis was performed. The stimulation paradigm was convolved with a hemodynamic response function (HRF) peaking at 1 sec. A one-tailed voxel-wise t-test was performed, tested for a minimum significance level of 0.001 with a minimum cluster size of 16 voxels and corrected for multiple comparisons using a cluster false discovery rate test (FDR).

For ROI analysis, for each animal, relevant subcortical anatomical ROIs (cochlear nucleus CN, superior olivary complex SOC, lateral lemniscus LL, inferior colliculus IC, medial geniculate body MGB) (Paxinos & Watson, 2009) were selected, depending on each specific experiment, for manual ROI delineation. The individual time courses were detrended with a fifth degree polynomial fit to the resting periods in order to remove low frequency trends, and then converted into percent signal change relative to baseline. For each run, individual cycles were separated and averaged across all animals to obtain the averaged response within each ROI (along with the standard error of the mean across animals). During binaural stimulation, as there is no meaningful contralateral or ipsilateral distinction, equivalent structures in both hemispheres show similar responses. Consequently, rather than limiting our analysis to a single hemisphere, we used a randomized subsample of all available acquisitions across both sides. This approach allowed us to maintain a representative dataset while ensuring the total number of runs remained comparable with those in the monaural stimulation condition. Data from both sides were checked for possible laterality bias and no significant differences were found (not shown). Area under the curve was calculated by computing the approximate integral of each BOLD response by trapezoidal integration. This approach was applied to each individual ROI time course to assess overall BOLD signal dynamics in response to the experimental stimuli.

All quantitative assessments of BOLD signal changes are based on time-course data extracted from manually defined ROIs, rather than being inferred from activation maps. This approach was chosen intentionally to avoid introducing bias that can arise from interpreting thresholded or color-coded activation patterns, which may vary depending on statistical thresholds, preprocessing pipelines, or visualization choices. Manual ROI delineation based off an atlas ensured anatomical consistency and reproducibility across subjects. ROIs were carefully placed based on identifiable anatomical landmarks, allowing for precise targeting of relevant auditory structures. By relying on atlas-based ROI placement rather than voxel-wise activation maps, we aimed to maintain objectivity and anatomical accuracy in our regional comparisons (regardless of normalization parameters), particularly when assessing subtle or bilateral effects in subcortical areas.

### 2.11 Statistical analysis

**Fig. 7.**
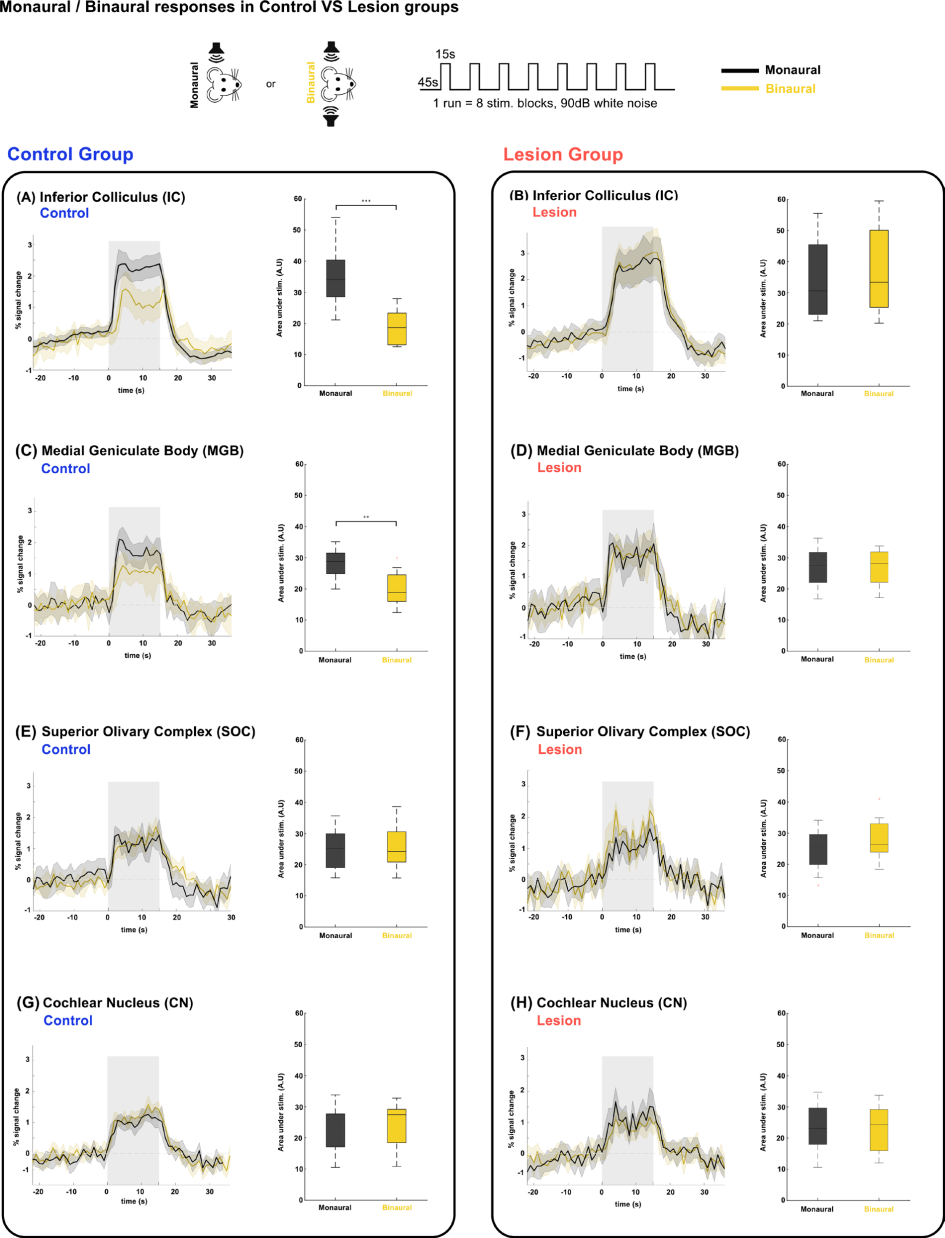
Auditory fMRI in monaural versus binaural stimulation. Plots show time courses of BOLD responses monaural/binaural stimulation of Control and Lesion groups. Translucid gray bars indicate the stimulation periods. Plots show the results of a two-sample parametric t-test, ∗p ≤ 0.05; ∗∗p ≤ 0.01; ∗∗∗p ≤ 0.001. N = 6 (8 slice acquisition) (A) IC in Control group, (B) IC in Lesion group, (C) MGB in Control, (D) MGB in Lesion group, (E) SOC in Control, (F) SOC in Lesion group, (G) CN in Control group, (H) CN in Lesion group. Binaural data use randomized subsample of all available acquisitions across both sides.

Data normality was confirmed with a one-sample Kolmogorov–Smirnov test at a 5% significance level. Unless otherwise specified, after correction for multiple comparisons, a p-value of 0.05 or less was considered statistically significant. For comparison of BOLD responses between control/lesion (Fig. 5) and monaural/binaural regimes (Fig. 7), a two-sample *t*-test was performed (∗p ≤ 0.05; ∗∗p ≤ 0.01; ∗∗∗p ≤ 0.001). For stimulus modulation comparison (ramped stimulus, Fig. 4) a non-parametric Kruskal–Wallis statistical test was performed (∗p ≤ 0.05; ∗∗p ≤ 0.01; ∗∗∗p ≤ 0.001), with post hoc analysis corrected for multiple comparisons.

### 2.12 Exclusion criteria

Of all the animals used in our experiments (N = 56), we excluded in total 13 animals, with 43 remaining as part of the study. Exclusion criteria were a priori determined as follows:

1. Animals that showed no BOLD response upon monaural stimulation after 75 min post-induction (N = 7) were removed from the scanner, the experiment was terminated, and the animal excluded from the study assuming either unstable physiological conditions for BOLD-fMRI (for example, abnormally high or low respiration rates, deviations in core body temperature) and/or a poor delivery of sound to the ears.
2. Acquisitions that displayed severe artifacts (e.g., ghosts) or tSNR <40 were excluded from analysis (N = 2).
3. In lesioned animals, the effectiveness of each lesion (IC, VC or sham) was verified post-surgery, both through anatomical MRI scans (determining the location and extent of lesions) and with fMRI acquisitions (targeting the demonstration of lack of activation within the lesioned structure). N = 2 animals did not meet the criteria for successful lesion induction and were, therefore, excluded. In addition, N = 2 animals experienced abnormal post-surgery conditions, such as excess swelling around the suture or visible lethargy, and were thus also excluded from the study.

## 3 Results

### 3.1 High-quality fMRI data along the subcortical auditory pathway

Raw data from a single run in a single representative animal are shown in Figure 2, for both contralateral and ipsilateral ICs. Figure 2A shows anatomical data, while the raw GE-EPI images corresponding to a single run of the paradigm, in a single animal, are shown in Figure 2B. At the individual animal level, tSNR (Murphy et al., 2007) in the key areas explored was 151 ± 18 in IC, 102 ± 32 in MGB, 85 ± 9 in SOC, and 73 ± 12 in CN. The BOLD responses—whether positive or negative—could be observed with the naked eye in the relevant auditory pathway ROIs even in single runs (Fig. 2C), before averaging on multiple runs or animals.

### 3.2 Monaural stimulation elicits strong positive/negative fMRI signals in cIC/iIC

When monaural stimulation was delivered to the rats, strong positive activation was observed in the cIC (Fig. 3A, warm colors), while, strikingly, strong negative fMRI responses were observed in the iIC (Fig. 3A, cool colors). The corresponding t-values in these areas were very large, reaching ~+32 and ~-19 in the cIC and iIC, respectively. In the rest of the pathway, robust positive BOLD responses were recorded in ipsilateral CN, and contralateral SOC, IC, LL, and MGB (Fig. 3A, warm colors). ROI analysis in predefined ROIs confirmed both the strong positive responses along the pathway and the negative responses in iIC. Supplementary Figure S3 shows data from both hemispheres, showing no activation on contralateral CN, and ipsilateral SOC and MGB. Supplementary Figure S4 shows additional cortical data, and Supplementary Figure S5 shows a comparison between medetomidine and isoflurane responses for the same experiment, showing comparable responses baring iIC lacking negative BOLD responses (NBRs).

### 3.3 cIC but not iIC responses track a ramped monaural stimulus

To investigate how the inferior colliculus in each hemisphere, contralateral and ipsilateral to the stimulated ear, differs in temporal response dynamics and sensitivity to amplitude changes, we employed amplitude-modulated auditory stimuli. Specifically, we used ramped, amplitude-modulated sounds (Fig. 4) designed to engage temporally integrative processes within the auditory pathway. The IC responses to these monaural stimuli (see envelopes in Supplementary Fig. S6) are shown in Figure 4. In cIC (Fig. 4A), the fMRI signals clearly track the ramped stimulus envelopes, with the “Early Rise” ramp (black) yielding the highest BOLD signal changes, while decreasing responses were observed for the Intermediate (green), and “Late Rise” (purple) ramp profiles. Moreover, quantitatively, a statistically significant difference between all three ramps was observed, not only during the entire stimulus duration, but also in the amplitude of the plateau.

In the iIC (Fig. 4B), the responses are notably distinct: ipsilateral negative responses exhibit dynamics that differ markedly from contralateral responses. They are initiated only when a certain intensity threshold is reached and then maintain a consistent profile thereafter. This is best seen with the “Early Rise” ramp response that evidences a relatively flat response for the entire duration of the stimulus regardless of the increasing amplitude modulation, with iIC responses depending on the slope of the ramp (how quickly the stimulus amplitude changes) rather than the amplitude of the stimulus. Furthermore, the amplitude plateau (last 5 sec of stimulation) revealed no differences between the three ramp modes. Another interesting feature of iIC activity is the post-stimulus response: a sharp positive fMRI signal that was consistently observed, reaching statistically significantly higher levels for the “Early Rise” ramp stimulus.

### 3.4 An intact IC is necessary for the “pull” interaction

To further investigate push–pull relationships between the ICs upon monaural stimulation, we modulated communication within the auditory system by unilaterally lesioning the IC (Fig. 5). Figure 5A shows structural T_2_-weighted MR images confirming the correct anatomical location and extent of the unilateral IC lesion via a single representative animal (white arrows point at the lesioned site) and corresponding histology. The lesion was well confined to the targeted area, though some degree of inflammation can be observed in the cerebellum, most likely due to the mechanical effects of the injection. This small inflammatory response subsided with time, unlike the damage in the IC lesions (data not shown). Importantly, these images also confirm that the IC on the opposite side was not damaged by the unilateral IC lesion procedure, and remained anatomically intact.

Our first objective was to confirm the functional preservation of the healthy, unlesioned IC. To assess this, we presented auditory stimulation to the ear ipsilateral to the lesioned site and examined the resulting BOLD response in the contralateral (i.e., intact) IC. Under normal physiological conditions, such stimulation is expected to elicit a robust positive BOLD response in the contralateral auditory pathway. In our lesioned model (Fig. 5B, in red), we observed that this positive BOLD response in the contralateral IC was clearly preserved, indicating that the lesion did not significantly disrupt the function of the auditory structures on the unaffected side. This finding is particularly important as it demonstrates that, despite the unilateral ablation of the IC, the auditory system continues to support normal contralateral processing.

Next, we aimed to confirm the effectiveness of the lesion. To achieve this, we presented auditory stimulation to the ear contralateral to the lesioned site, which under normal conditions would strongly activate the corresponding IC. We then examined the BOLD time-course response within the ROI encompassing the lesioned area.

As anticipated, no detectable BOLD fMRI signal was observed in the lesioned group, confirming the functional inactivation of the targeted IC (Fig. 5C, in red). This absence of response supports the effectiveness of the lesion in disrupting auditory processing in the affected structure. In contrast, animals in the control group, which did not undergo any lesioning, exhibited the expected robust positive BOLD responses in the corresponding IC (Fig. 5C, in blue), with the differences between control and lesioned animals reaching very high statistically significant levels.

Finally, we sought to investigate the impact of our lesion model on the presence of ipsilateral negative BOLD responses, a key feature of the push–pull dynamics observed in the auditory system. To do this, we once again presented auditory stimulation to the ear contralateral to the lesioned side, but this time focused our analysis on the ipsilateral (healthy) side of the brain.

In control animals, as expected, this paradigm elicited a robust negative BOLD response in the IC ipsilateral to the stimulus, consistent with previous observations (Fig. 5D, in blue). However, in the lesioned group, this negative response was completely abolished, despite the fact that the region under observation was anatomically intact and unlesioned (Fig. 5D, in red). This finding is particularly striking, as it suggests that the effects of a unilateral IC lesion extend beyond the site of injury and disrupt functional activity in the opposite hemisphere.

Note that a sharp positive signal at the end of the stimulation (also seen in the Control group) was also observed. Taken together, these results show the necessity of an intact IC for the observation of negative BOLD responses. To ensure the specificity of our findings and rule out non-specific effects of the surgical procedure itself, we also conducted sham surgeries (saline injected into IC), as well as control lesions targeted to the visual cortex (VC). As shown in Supplementary Figure S7, both of these control conditions preserved the typical pattern of auditory-evoked BOLD responses in the IC.

### 3.5 Push–pull interactions in binaural stimulation reduce activity compared with monaural stimulation

Figure 6 shows fMRI responses to binaural stimulation in healthy subjects, where each colliculus serves both as ipsilateral and contralateral to the stimulation from each ear. Spatially, the binaural stimulus elicited a fairly symmetrical positive activation pattern in CN, SOC, LL, IC, and MGB in both hemispheres. Unlike its monaural counterpart, no negative responses were observed upon binaural stimulation. The fMRI time courses upon monaural stimulation in ROIs placed in CN, SOC, IC, and MGB are shown in Figure 6B, where the robustness of the positive responses and the absence of any negative responses are confirmed.

Given all the observations above, we hypothesized that the push–pull mechanism is still present in binaural stimulation. In particular, if the ipsilateral inferior colliculi produced negative responses to ipsilateral monaural stimulation, the (positive) activation in binaural stimulation should be decreased due to summation of this effect and the positive contralateral response. Figure 7 shows a comparison between monaural and binaural stimulation. The IC data shown in Figure 7A reveal that indeed binaural stimulation produces weaker (~30% lower) BOLD responses compared with monaural stimuli. Furthermore, Figure 7B shows that if one of the IC is lesioned, the “pull” effect is released, leading to responses of comparable amplitude (not statistically significantly different) between monaural and binaural stimulation. To account for the relatively high-sound amplitude and this effect being a purely a loudness regulating mechanism, the experiment was repeated at 60 dB, with Supplementary Figure S8 showing similar results in IC.

### 3.6 The push–pull interaction is not observed in structures earlier than IC, and is relayed to MGB

We further investigated the responses of relevant structures upstream or downstream of IC. Responses in MGB, SOC, and CN are shown in Figure 7C–H. Notably, the same pattern as observed in IC can be seen in the MGB (decrease in amplitude for binaural stimulation), while fMRI responses in the earlier structures in the ascending auditory pathway, such as the SOC (Fig. 7E, F) and CN (Fig. 7G, H), seem unaffected when comparing monaural/binaural stimulations in both control and lesion groups. No statistically significant differences were observed between left and right structures upon binaural stimulation. Interestingly, the MGB does not show NBRs upon monaural stimulation, and only seems to reproduce the result of the IC push-pull, further reinforcing its role as a relay structure (Mei & Chen, 2010) between IC and AC, and not as the origin of this mechanism. We note in passing that despite some slight SNR reduction in some operated animals due to post-surgical fluid accumulation above the brain, SNR was sufficient to infer the differences.

## 4 Discussion

Activity in subcortical areas is critical for many aspects of auditory processing (Casseday et al., 2002; Kavanagh & Kelly, 1992; Malmierca, 2006). A push–pull mechanism in the IC, previously demonstrated by electrophysiology (Liu et al., 2022; Xiong et al., 2013), is thought to be involved in processes of sound source localization and discrimination. Here, we asked whether this kind of interaction can be observed with fMRI, and how it interacts at the pathway-wide level. Our main findings include the first (to our knowledge) observation of negative BOLD signals in the rat ipsilateral inferior colliculus following monaural stimulation, accompanied by positive BOLD signals in the contralateral IC. This pattern reflects a population-level push–pull mechanism as seen by BOLD fMRI. We further demonstrate that these push–pull dynamics persist during binaural stimulation and depend on the structural and functional integrity of the colliculi. Our data also show that the push–pull interaction, at least at the population level represented by BOLD fMRI, originates in IC and not in earlier structures, such as SOC, and that the push–pull consequence is then relayed downstream to the MGB. Our findings point to a major role for both ICs in sound processing and source localization and reinforce the importance of subcortical structures and their interactions in the auditory pathway. Below (and in the Supplementary Discussion), we discuss each of these aspects in more detail.

### 4.1 Negative BOLD in ipsilateral IC

Our results evidenced strong positive BOLD in subcortical auditory pathway regions of the ipsilateral CN, and contralateral IC, SOC, LL, and MGB. Although BOLD responses in these regions had been previously reported (Cheung et al., 2012) and shown to align with electrophysiological evidence (H. J. Lee et al., 2012; Malmierca, 2015), our study is the first to observe negative BOLD responses in the iIC (Fig. 3B). This negative BOLD response likely reflects the known inhibitory responses seen in ipsilateral responses in IC (Hind et al., 1963; Li & Kelly, 1992). A recent study on LFPs and MUAs in the adjacent superior colliculus has shown strong deactivation of the areas tied to negative BOLD responses during rapid visual stimulation (Gil, Valente, & Shemesh, 2024). This deactivation is likely due to inhibition, aligning with previous research suggesting that negative BOLD signals primarily result from inhibitory processes (Devor et al., 2007; Shmuel et al., 2006; Sten et al., 2017). An alternative, or complementary, explanation for the observed negative BOLD seen in the ipsilateral IC could also involve a vascular steal phenomenon (Wade, 2002). In this context, rather than reflecting active neural inhibition alone, the negative BOLD signal may partially result from localized reductions in CBF due to the redistribution of vascular resources. During strong unilateral activation, as in the contralateral IC, regional increases in blood flow could effectively “steal” perfusion from neighboring or functionally connected regions, leading to a relative drop in oxygenated blood supply, and thus a negative BOLD signal on the ipsilateral side. We do, however, consider vascular steal an unlikely explanation for the majority of the effects observed in our study for several key reasons. The absence of negative BOLD responses in the lesioned animals, despite preserved positive responses in the contralateral IC, suggests that the mechanism responsible for the ipsilateral negative BOLD signal is dependent on intact intercollicular pathway, rather than on hemodynamic competition. If vascular steal were the primary mechanism, one would expect that the removal of one IC would continue to produce (to some degree) a relative drop in perfusion to the opposite hemisphere. Instead, we observe the complete abolition of the negative BOLD response, which points to a neural origin rather than a vascular one. More so, the absence of similar negative BOLD patterns in other auditory structures besides iIC (particularly the ipsilateral MGB) also shows the specificity of the effect. Furthermore, a recent similar experiment in visual responses at the level of the superior colliculus in rats (Gil, Valente, & Shemesh, 2024) has shown strong correlations between MUA and NBRs, strongly pointing to neuronal suppression as the most probable cause for these negative responses.

It is important to address the discrepancies between our findings and previously published studies on auditory fMRI in rodents (Cheung et al., 2012; Lau et al., 2013; Zhang et al., 2013), particularly in the presence of ipsilateral negative BOLD responses in iIC, while most contralateral structures have shown positive activation in line with the literature. Our main hypothesis on this discrepancy is that the anesthetic regimes (medetomidine vs. isoflurane) and perhaps sensitivity played a crucial factor. Anesthetics have been shown to affect both brain states (Paasonen et al., 2018) and BOLD signals, potentially masking negative BOLD. This is further supported by our Supplementary Figure S5, where we replicated the lack of negative BOLD upon a monaural stimulation paradigm with animals under light isoflurane anesthesia (~1.5%), as in Cheung et al. (2012). Under this regime, most subcortical structures exhibited similar results to those under medetomidine sedation except for the negative BOLD responses in iIC that were absent in the isoflurane group. Thus, it is most likely that the medetomidine used here allowed for the detection of this effect.

This discrepancy in responses was also one of the key motivations behind performing sham surgeries and control lesions in non-auditory regions (VC). Our goal was to determine whether the occurrence and subsequent abolition of negative BOLD responses observed in iIC following unilateral IC lesions could be attributed to factors unrelated to the lesion itself, such as non-specific surgical effects or the use of medetomidine anesthesia. Importantly, in both the sham group and VC lesion group, we continued to observe robust negative BOLD responses in iIC (Supplementary Fig. S7), similar to those seen in non-lesioned control animals. All groups, including the lesioned, sham, and control animals, were scanned under identical medetomidine sedation protocols, ensuring consistent anesthetic conditions across experiments. The fact that negative BOLD responses are specifically abolished only in animals with targeted IC lesions, and not in those with sham or unrelated cortical lesions, strongly suggests that the effect is not a consequence of medetomidine-induced vascular changes, but rather reflects a true neural or neurovascular response dependent on the integrity of the inferior colliculus.

Taken together, these observations strongly suggest that the negative BOLD signals observed in the ipsilateral IC are not primarily artifacts of vascular steal or the anesthetic agent but instead reflect active, stimulus-driven suppression mechanisms that are disrupted following unilateral lesioning.

### 4.2 Ipsi/Contralateral IC responses exhibit different dynamics

We further probed the relationship between the two IC responses by presenting a time-dependent varying amplitude ramped stimulus (Fig. 4). Our finding of distinct ramped signals and plateaus only in the cIC likely indicates a lasting temporally integrative mechanism, since all three ramps reached 90 dB after 25 sec and plateaued for the last 5 sec. The differences in how the IC responds to different ramp stimuli could reflect both a time-dependent integration (Gans et al., 2009) and a faster habituation to the “Late Rise” ramp stimulus (Fox, 1979; Prado-Gutierrez et al., 2015), as it can be initially perceived as a long, almost continuous stimulus due to its slow initial variation in amplitude. However, the iIC response emerged later than the cIC response, and exhibited an on/off behavior, regardless of stimulation envelope profile. We hypothesize that the ipsilateral responses are evoked with a higher stimulus amplitude threshold (Semple & Kitzes, 1985) (n.b. that the “Late Rise” ramp stimulus shows a nearly flat iIC response until >~20 sec after the stimulus onset), and that they depend on cIC signaling (as seen in the Lesion group in Fig. 5).

Another interesting feature of the iIC signals is the post-stimulus positive response, seen in all three ramp paradigms. Our interpretation, which requires further validation in future studies, is that this sharp signal likely represents an “offset response”—a brief activation of neurons signaling the end of stimulus (Kasai et al., 2012; Solyga & Barkat, 2021). However, when compared with the more consistent positive and negative responses observed during stimulus presentation, the post-stimulus activity exhibited some inter-animal (and inter-paradigm) variability, with some animals showing a very clear positive peak right after activation, while others displayed a more subdued response. This variability made it difficult to draw direct conclusions about the underlying mechanisms of the overshoot, and we, therefore, prioritized analyzing the more robust stimulus-locked components of the response.

Ultimately, the iIC/cIC responses are clearly not a mirrored image of each other. Ipsilateral responses in IC have been shown to differ from their contralateral counterparts on single unit (Semple & Kitzes, 1985) and population levels (Klug et al., 1999; Ping et al., 2008), using pure tones and noise bursts. Such differences are further corroborated here with our results reinforcing and expanding on it by showing how they can be modulated using varying stimuli.

It is important to note that the three types of ramps used in our study differ not only in their envelope, but also other physical characteristics, including onset times, slopes, and total energy. Although these variables were not explicitly controlled for, it is plausible that each contributes uniquely to the observed neural responses. Future research could benefit from systematically isolating and manipulating these features to better understand their individual roles in shaping auditory processing.

### 4.3 IC as the origin for the BOLD push–pull mechanism

Knowing that the IC is a main integration hub of the auditory pathway, and with our own data and previous studies (Ito, 2020; Liu et al., 2022; Xiong et al., 2013) suggesting strong intercollicular communication, it seemed a promising structure for the origin of the push–pull mechanism, and thus we decided to use a unilateral IC lesion model to modulate this system. Our Lesion model results (Fig. 5) suggest two main points: First is the apparent preservation of contralateral monaural response in the healthy IC of the Lesion group, showing similar results to those of the control group. Second is the abolishment of ipsilateral negative BOLD responses. Because this is a structure that has not been physically compromised by the lesions on the opposite side of the brain, this indicates that the lesioned colliculus is (directly or indirectly) responsible for the ipsilateral negative response, potentially through intercollicular inhibitory/excitatory interactions, suggesting the origin of this mechanism to be the IC itself, further evidenced by the absence of such mechanism in earlier structures such as CN or SOC in Figure 7. While the exact nature of these interactions’ merits future investigation with, for example, electrophysiology, our unilateral IC Lesion model unequivocally demonstrates the necessity of collicular integrity for the ipsilateral negative BOLD responses, and thus, the push–pull mechanism (Fig. 1C). A plausible hypothesis is that this mechanism is a result of direct communication between ICs, through intercollicular projections via the commissure of the inferior colliculi (Aitkin & Phillips, 1984; Orton & Rees, 2014; Saldaña & Merchán, 1992), as the stimulation of this structure has been shown to be able to produce both excitatory/inhibitory responses on IC neurons (Ito et al., 2016; Malmierca et al., 2005) and being the closest relevant structure in the pathway. However, we note that the IC is a massively interconnected hub of integration (Malmierca, 2006), with both excitatory and inhibitory projections to most auditory structures, making it more difficult to pinpoint an exact path for this effect, from our study alone. In addition, as an anatomical simplification, we considered each auditory structure as a homogeneous area, a simplified view, in particular of such a large and interconnected structure like the IC. Studies using loose-patch recordings (Liu et al., 2022) have shown differences in responses between the central nucleus (ICC) and dorsal nucleus of the inferior colliculus (ICD), in which ICD neurons exhibited stronger responses to ipsilateral sound stimulation and better binaural summation than those of ICC neurons, pointing to greater heterogeneity within each IC. Perhaps specific region-focused data acquisition, with higher temporal/spatial resolution, could provide more information on specific areas within the auditory pathway structures.

Nevertheless, these positive/negative BOLD collicular dynamics upon monaural stimulation led us to the hypothesis that the result of binaural stimulation (Fig. 6) would not onlycomprise each individual positive contralateral response, but rather the summation of both positive and negative responses, effectively comprising a BOLD collicular push–pull mechanism. Furthermore, our hypothesis suggested that these binaural dynamics would be decreased by unilaterally lesioning IC due to the cessation of intercollicular signaling. Importantly, the unilateral lesion of the IC may disrupt this delicate balance, not only by eliminating the neural substrate responsible for generating local BOLD responses, but also by altering the systemic vascular dynamics of the network.

### 4.4 The BOLD push–pull mechanism in binaural stimulation

Our comparison between monaural and binaural stimulation in Control animals (Fig. 7A) showed that, in IC, binaural stimulation does in fact yield lower BOLD responses than monaural stimulation at the same intensity. In the monaural regime, there is no competition for auditory processing, as only one sound source is present in one of the ears. In contrast, we show the result of a binaural stimulation not only comprises each individual positive contralateral response but rather the summation of both positive and negative responses (Fig. 1C). This competition seems to drive each colliculus’s BOLD responses downward, which indicates that the mechanism generating negative BOLD with monaural stimulation is still present during binaural stimulation. Previous studies on subcortical ILD processing in rats (Lau et al., 2013) did not find these dynamics, likely due to the anesthetic regime (isoflurane) either masking or disrupting collicular function, and/or because of the paradigm design, where for each ILD setting, both the left and right ear volumes were adjusted by equal and opposite amounts instead of having a fixed value on one side and varying the other. This design produces a varying BOLD response each time, making it impossible to compare responses on both sides, and thus not reporting this lowered BOLD response for binaural stimulation, or any NBRs altogether. Another study in macaques (Ortiz-Rios et al., 2017) has shown, in both awake and anesthetized states, BOLD responses having an overall suppression effect to sound sources on the ipsilateral side on both AC and IC. Furthermore, it shows ipsilateral PBRs in AC were greatly reduced in size and accompanied by an NBR pattern in anterior and posterior regions, while IC showed no NBRs, only a lowered response to ipsilateral stimuli. However, these responses were elicited with more complex dynamic stimuli.

Previous studies using electrophysiology have shown that several structures of the central auditory system and a majority of IC neurons can be excited by contralateral sound input and suppressed by ipsilateral input (Casseday et al., 2002; Pollak, 2012), with responses to binaural stimulation being smaller than the summed response to monaural stimulation (Laumen et al., 2016). The ipsilateral suppression of responses to contralateral stimulation as a manner of gain control has also been suggested, possibly through MGB sending feedback inputs to the ipsilateral IC (Kuwabara & Zook, 2000; Malmierca et al., 2005), or intercollicular projections via the commissure of the inferior colliculi (CoIC) (Aitkin & Phillips, 1984; Orton & Rees, 2014; Saldaña & Merchán, 1992), with stimulation of the CoIC producing both excitatory/inhibitory effects on IC neurons. Earlier work on bilateral stimulation of the IC (Hind et al., 1963) had already suggested that simultaneous stimulation of both ears could produce spike counts that are significantly lower than those obtained when the excitatory ear alone is stimulated, with this excitatory/inhibitory interplay further confirmed as a push–pull mechanism (Xiong et al., 2013) by showing stronger contralateral excitation and relatively stronger ipsilateral inhibition in IC neurons, and excitatory inputs suggested as being altogether responsible for ipsilateral and binaural summation responses on IC (Liu et al., 2022). Our work is consistent with these earlier findings and adds a disruption to the system via unilaterally lesioning the IC. Our data from Lesion animals (Fig. 7B) show that monaural/binaural differences are abolished, confirming that unilaterally lesioning IC prevents this BOLD push–pull mechanism from exerting its influence. Of all the other structures studied, only MGB showed a decrease in signals from binaural to monaural stimulus, suggesting a similar push–pull mechanism may exist in the MGB. Similarly, these differences in MGB were abolished in the Lesion group. This is particularly interesting because unlike the ipsilateral IC response, MGB does not show any negative BOLD response to monaural stimulation (Fig. 3). We excluded the effects of loud stimulus amplitudes, which could be close to a saturation point for IC processing (Flores et al., 2015; Sheppard et al., 2017), by repeating the experiments with a lower amplitude (Supplementary Fig. S8) at 60 dB, which yielded similar results in the IC. It is important to mention that partial volume effects between the IC and MGB can potentially play a role in these signals, given the anatomical proximity of the structures. To mitigate this potential spatial overlap, we avoided analyzing the most posterior slice of MGB, where IC and MGB voxels may contain partial volume from each other, during ROI analysis. Furthermore, the ipsilateral MGB shows no BOLD activation while the iIC exhibits negative BOLD in the same experiment, which gives us some confidence that the observed MGB activation is not arising from IC contamination, and mainly reflects true MGB involvement.

### 4.5 fMRI as a window into excitatory–inhibitory circuit dynamics

In summary, our work presents a dissection of subcortical interactions via advanced fMRI and lesions in the rat, including first evidence for a push–pull mechanism in BOLD signals, which originates in IC and is relayed to MGB. These findings highlight the importance of subcortical interactions in auditory processing and lay the foundation for deeper investigations of push–pull effects using fMRI. Although we cannot claim with absolute certainty that the push–pull BOLD mechanism observed in our study is a direct one-to-one match with the inhibitory–excitatory dynamics reported in the electrophysiological literature, the resemblance is nonetheless compelling. Our data suggest that the BOLD signal, despite its indirect nature, may be capturing a large-scale manifestation of the same underlying circuit dynamics, namely, the spatially and temporally coordinated interplay between excitation and inhibition within the inferior colliculus and its ramifications along the auditory pathway. The fact that the BOLD responses in our experiments follow patterns that align so closely with known neuronal response profiles provides strong evidence for a shared mechanism. In this context, the BOLD signal can be interpreted not merely as a general indicator of neural activity, but also as a potentially rich, albeit smoothed, readout of specific population-level interactions. The push–pull dynamics we observe, with regions showing reciprocal increases and decreases in signal, mirror the finely tuned balance of excitation and inhibition observed in invasive electrophysiological studies, reinforcing the idea that functional MRI can offer insights into circuit-level processing. This alignment between imaging and electrophysiology strengthens the case for using fMRI, especially at high resolution, to infer not just where activity is occurring, but also how different neural populations may be interacting.

## Supplementary Material

Supplementary Material

## Data Availability

The raw structural and functional MR images and the Matlab code used in this study are available for download at https://figshare.com/articles/dataset/Fred_Severo_etal_datasets/29974120?file=57385204.
