# Supplementary material for: Functional MRI reveals subcortical auditory push–pull interactions requiring intercollicular integrity
Source: Imaging Neurosci (Camb). 2025 Sep 22;3:IMAG.a.155. doi: 10.1162/IMAG.a.155 (PMC12455056; doi:10.1162/IMAG.a.155)
Supplement: Supplementary Material [file IMAG.a.155_supp.pdf]

Supplementary Information

for

Functional MRI reveals subcortical auditory push-pull interactions requiring intercollicular integrity

Frederico Severo, Mafalda Valente, Noam Shemesh

Champalimaud Research, Champalimaud Foundation, Lisbon PT

*Corresponding author:

Dr. Noam Shemesh, Champalimaud Research, Champalimaud Foundation, Av. Brasilia 1400-038, Lisbon, Portugal

E-mail: noam.shemesh@neuro.fchampalimaud.org

Conflict of Interest: NS serves on the Bruker Biospin scientific advisory board.

Abbreviated title: Auditory push/pull interactions rely on intercollicular integrity

Acknowledgements

This study was funded in part by the European Research Council (agreement No. 679058), as well as by Fundação para a Ciência e Tecnologia (project 275-FCT PTDC/BBB IMG/5132/2014. The authors acknowledge the vivarium of the Champalimaud Centre for the Unknown, a facility of CONGENTO which is a research infrastructure co financed by Lisboa Regional Operational Programme (Lisboa 2020), under the PORTUGAL 2020 Partnership Agreement through the European Regional Development Fund and Fundação para a Ciência e Tecnologia (project LISBOA 01 0145 FEDER 022170). FS thanks Fundação para a Ciência e Tecnologia for a PhD fellowship PD/BD/141648/2018, and MV thanks Fundação para a Ciência e Tecnologia for a PhD fellowship PD/BD/141560/2018. All authors would like to thank Dr. Cristina Chavarrías for implementing the fMRI triggering, Ms. Francisca F Fernandes for customized fMRI analysis Matlab code, and Dr. Rita Gil and Dr. Joana Carvalho for insightful discussions on the project.

Supplementary Discussion

Differences due to anesthetic regimes

As mentioned, previous studies on auditory fMRI in rats have mostly been conducted under isoflurane (Cheung et al., 2012; Lau et al., 2013; Zhang et al., 2013) rather than medetomidine. Auditory discrimination in rats has been shown to be affected even at extremely low doses of isoflurane (0.2 to 0.4%), showing decreased sensory efficiency (Burlingame et al., 2007) as well as changes in latency and amplitude of auditory responses (Bielefeld, 2014). Isoflurane has also been linked to poor resting state functional connectivity (Xie et al., 2020) and decreased spontaneous neural activity (van Alst et al., 2019) and its known mechanism for vasodilation (Schwinn et al., 1990), coupled with a close link between neuronal inhibition and arteriolar vasoconstriction corresponding to a decrease in blood oxygenation (Devor et al., 2007), supports our hypothesis of anesthesia regime discrepancy, and why positive BOLD responses persist under isoflurane, but negative responses are no longer present (as per Fig.S5). Conversely, light sedation using medetomidine has been shown to preserve connectivity networks in a greater level of detail (Kalthoff et al., 2013), and may therefore be considered superior to standard isoflurane anesthesia.

Post Stimulus Response

A point of interest that was not fully addressed in the main discussion is the sharp post stimulus positive response seen in the healthy ipsilateral IC upon monaural stimulation, as it is still present after the unilateral IC lesions (Fig.5B), suggesting that it may have a different origin altogether. A possible explanation is that it represents an offset response (Kasai et al., 2012; Solyga & Barkat, 2021), which appears after a sound terminates. However, the neural mechanisms that evoke these offset responses are not well understood. A similar post stimulus BOLD overshoot was also reported in the superior colliculus, and further corroborated with electrophysiology, in a visual stimulation context (Gil, Valente, & Shemesh, 2024), suggesting some similarities on how both collicular structures respond to the termination of a stimulus, regardless of its modality. The IC is also known to be an integral part of

deviancy and novelty detection in the continuous flow of auditory information (Aguilar Ayala & Malmierca, 2013; Zhao et al., 2011). The end of the white noise stimulus may cause the underlying scanner noise to become more salient and therefore trigger this response.

Lesion model controls

Sham lesions (unilateral saline injections in IC) and Visual Cortex lesions (with ibotenic acid) were also performed (Fig.S7), to exclude effects of the surgery itself, and global effects of the ibotenic acid. VC coordinates for the craniotomies and number of injections required were determined for each individual animal based on T_2-weighted anatomical images acquired before the surgery. Injections were made in a maximum of 5 different Anterior-Posterior (AP) coordinates, with 1 injection site for the first AP coordinate (2 pulses of injection) and 2 for the following (4 pulses of injection each), for full coverage of the V1. Both controls did not abolish ipsilateral negative BOLD in monaural stimulation, suggesting this negative signal is only abolished when the IC as a whole is incapable of responding to the stimulus. These animals also acted as controls for prior isoflurane exposure, as, regardless of these animals having been exposed to isoflurane during surgery planning and surgery itself a day before scanning, they still evidenced clear negative BOLD responses upon monaural stimulation.

ILD vs ITD

While we focused on a strict monaural/binaural stimulation as a conduit for ILDs, and discarded ITDs as an important binaural cue, rats are still sensitive to timing and phase differences in sound. Even if they don't appear relevant to sound localization, ITD sensitive neurons exist in the rodent IC (Batra et al., 1993), despite their lack of low-frequency hearing, and their sensitivity has been linked to the binaural interaction of excitation and inhibition in the lateral superior olive (Ono & Ito, 2018), with some studies suggesting a shared mechanism operating across ILD and ITD localization cues (Orton et al., 2016). Similarly, assuming the binaural response to be a simple summation of a positive contralateral response and a negative ipsilateral one is rather reductive, as monaural and binaural responses have been shown

to have their own specific dynamics (Liu et al., 2022; Wei et al., 2018), with IC neurons not simply mediated by the summation of the inputs evoked by ipsilateral and contralateral stimulations, suggesting additional integration of acoustic information at the brainstem level.

Role of Auditory Cortex

The extent of the functional role of the auditory cortex and the corticofugal descending projections should also be addressed, particularly in the context of rodent models. Previous auditory fMRI data in rodents has given very little relevant information on auditory cortex, either showing activity in AC in rats to be the smallest of all the relevant structures (Cheung et al., 2012; Zhang et al., 2013) as well as our own data Fig.S4), or being completely absent in mice (Blazquez Freches et al., 2018). Several factors could possibly explain this. The relevance and complexity of the stimulus, as white noise is used in this study as a task-irrelevant auditory input without temporal structure, could be a reason. It has been suggested that AC responses and corticofugal descending connections have an important role in particularly challenging, behaviorally meaningful situations (Souffi et al., 2021), while the discriminative ability of subcortical neurons may be sufficient in most simple acoustic situations, as is the case here. Additionally, in the rat, information about stimulus identity is progressively sparser going from IC, to MGB, to AC, for spike counts, latency and temporal spiking patterns (Chechik et al., 2006), discrimination abilities of collicular and thalamic neurons are reported to fare better than those of cortical neurons (Souffi et al., 2021), while information such as the physical attributes of the stimulus and the animals' behavior can be decoded from the activity of subcortical neurons alone with a high degree of accuracy (T.-Y. Lee et al., 2023). Furthermore, the auditory cortex was found not to be at all essential for discrimination of the spatial locations of auditory stimuli (Kelly & Glazier, 1978), and subcortical responses can remain mostly unaffected during cortical inactivation (Cotillon & Edeline, 2000). Different anesthetics have also been linked both to a decrease (Bielefeld, 2014) and increase (Huang et al., 2022) in auditory sensitivity, and auditory cortex neurons from rats receiving medetomidine anesthesia showed enhanced inhibition and

low intrinsic excitability (Osanai & Tateno, 2016). Another factor is the habituation to the scanner noise throughout a session. Cortical structures have shown to be particularly sensitive to habituation, with decreased evoked potentials (Cook et al., 1968; Rosburg et al., 2006; Westenberg & Weinberger, 1976), lower BOLD responses (Klingner et al., 2011; Poellinger et al., 2001; Rabe et al., 2006), and a decrease in synaptic activity (Wilson, 1998). While these scanner sounds are repetitive in nature and only encompass a fraction of our frequency range of stimulation (Fig.S2), mostly on the lower end of the frequency spectrum, our auditory stimulation is undoubtedly being presented over this constant, recurring background noise, altering the amplitude threshold at which the animal will be able to perceive the presented stimuli, as previous studies have shown that constant binaural background noise can result in both enhancement as well as suppression of responses upon overlaid sounds (Lui et al., 2015).

Role of MGB

The MGB is a complex of nuclei that receive massive input from subcortical structures and thus serves as a major synaptic station in the pathways for information reaching auditory areas of the cerebral cortex(Brugge & Howard, 2002). It does, in fact, receive most of its anatomic input from the central nucleus of the IC (Eliades & Tsunada, 2019), acting as a relay between subcortical and cortical structures in the auditory pathway. The fact that it shares similar responses to IC in this BOLD push-pull mechanism further suggests how closely linked these two structures are. While the IC is thought of as the first level at which integrative processes execute functions akin to cognitive processing (Miller & Covey, 2011), it is not the only structure that has exhibited this kind of capabilities, as the MGB has been shown to be capable of frequency analysis (Bartlett et al., 2011), integration and processing of intensity and latency (Gil-Loyzaga, 2010), and also exhibits differential sensitivity to binaural and monaural spectral cues (Altman et al., 1970; Samson et al., 2000). However, it has also been reported that information about stimulus identity is progressively reduced in single MGB neurons (and then AC) relative to single IC neurons, when information is measured using spike counts, latency, or temporal spiking patterns

(Chechik et al., 2006). On the other hand, IC neurons are substantially more redundant than MGB neurons, largely due to increased frequency and spatial selectivity, likely because of its role as the first main processing hub of auditory stimulus.

Other auditory pathway structures

In earlier structures in the ascending auditory pathway, we show that SOC (Fig.7F) and CN (Fig.7H) responses are, unlike MGB, not influenced by both monaural/binaural stimulations and IC lesions, suggesting that feedforward projections from IC could play a bigger role when compared in auditory processing to feedback projections from IC. Previous studies have shown that lesions in IC can have long term anatomical plasticity effects in SOC (Okoyama et al., 1995), but as we performed these experiments roughly 24 h post lesion these effects are expected to be negligible. Conversely, SOC (Sally & Kelly, 1992) lesions have shown remarkably little effect on IC, where binaural summation and suppression responses were mostly unchanged following bilateral lesions in SOC. Thus, our hypothesis is that the BOLD push/pull mechanism we see in IC (and is further relayed to MGB) results from direct collicular processing and intercollicular communication that is then passed on to MGB through feedforward projections. Nevertheless, the contribution of other auditory structures in monaural and binaural processing in IC cannot be understated. Previous studies have shown the predominantly inhibitory influence of LL (Li & Kelly, 1992), SOC (Greene & Davis, 2019), and CN (Davis, 2002) on auditory responses in IC upon reversible blocking of the excitatory activity in these structures, where modulation in IC is mostly shown contralaterally.

Supplementary Figures and Captions

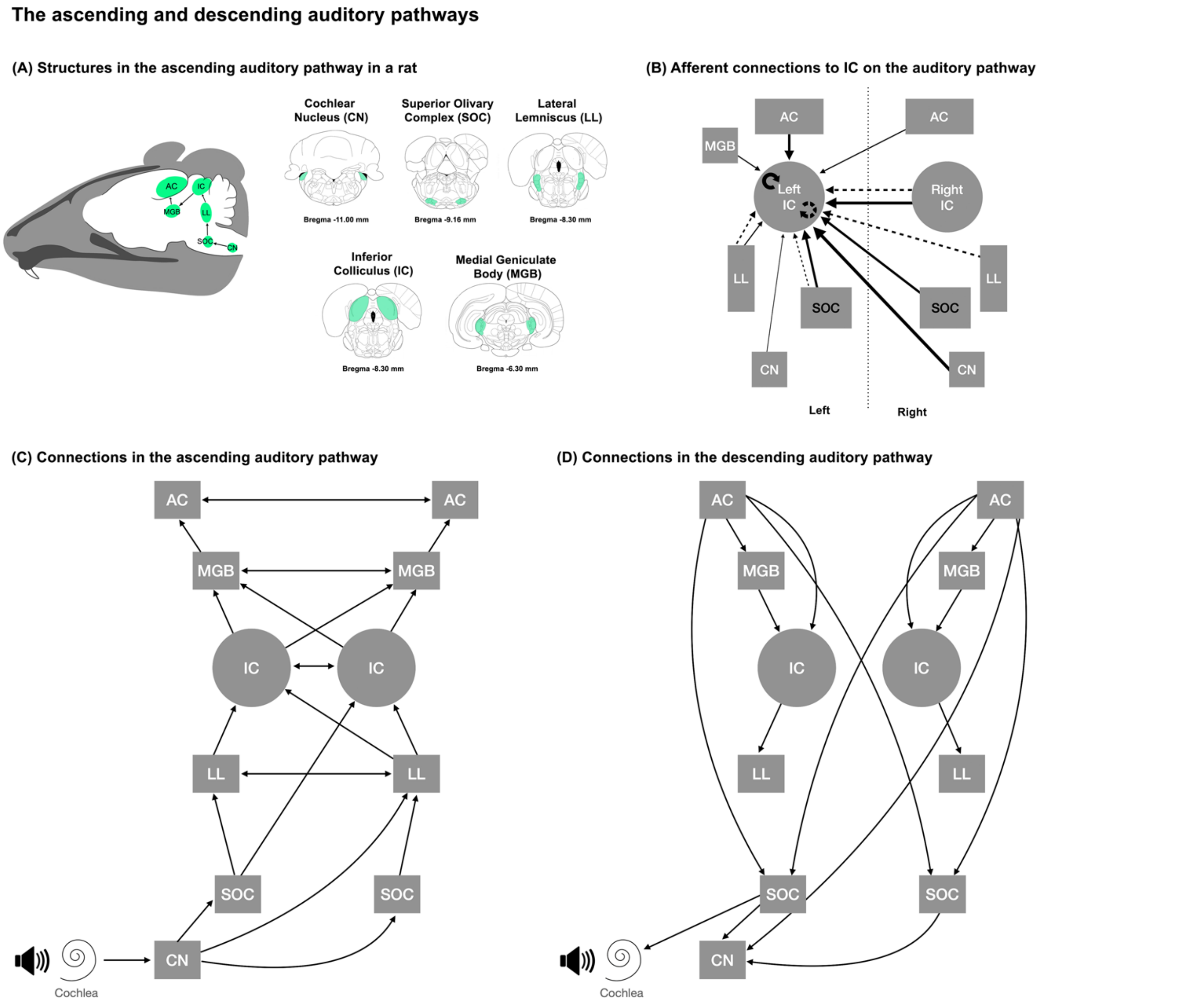

Fig.S1 (A) Structures in the subcortical ascending auditory pathway in a rat, Cochlear Nucleus (CN), Superior Olivary Complex (SOC), Lateral Lemniscus (LL), Inferior Colliculus (IC) and Medial Geniculate Body (MGB) **(B)** Schematic diagram of the excitatory (solid) and inhibitory (dashed) projections to the inferior colliculus. The thickness of the lines denotes the relative strength of the inhibitory and excitatory projections. The vertical dotted line indicates the midline. **(C)** Connections in the ascending auditory pathway **(D)** Connections in the descending auditory pathway (Malmierca, 2015)

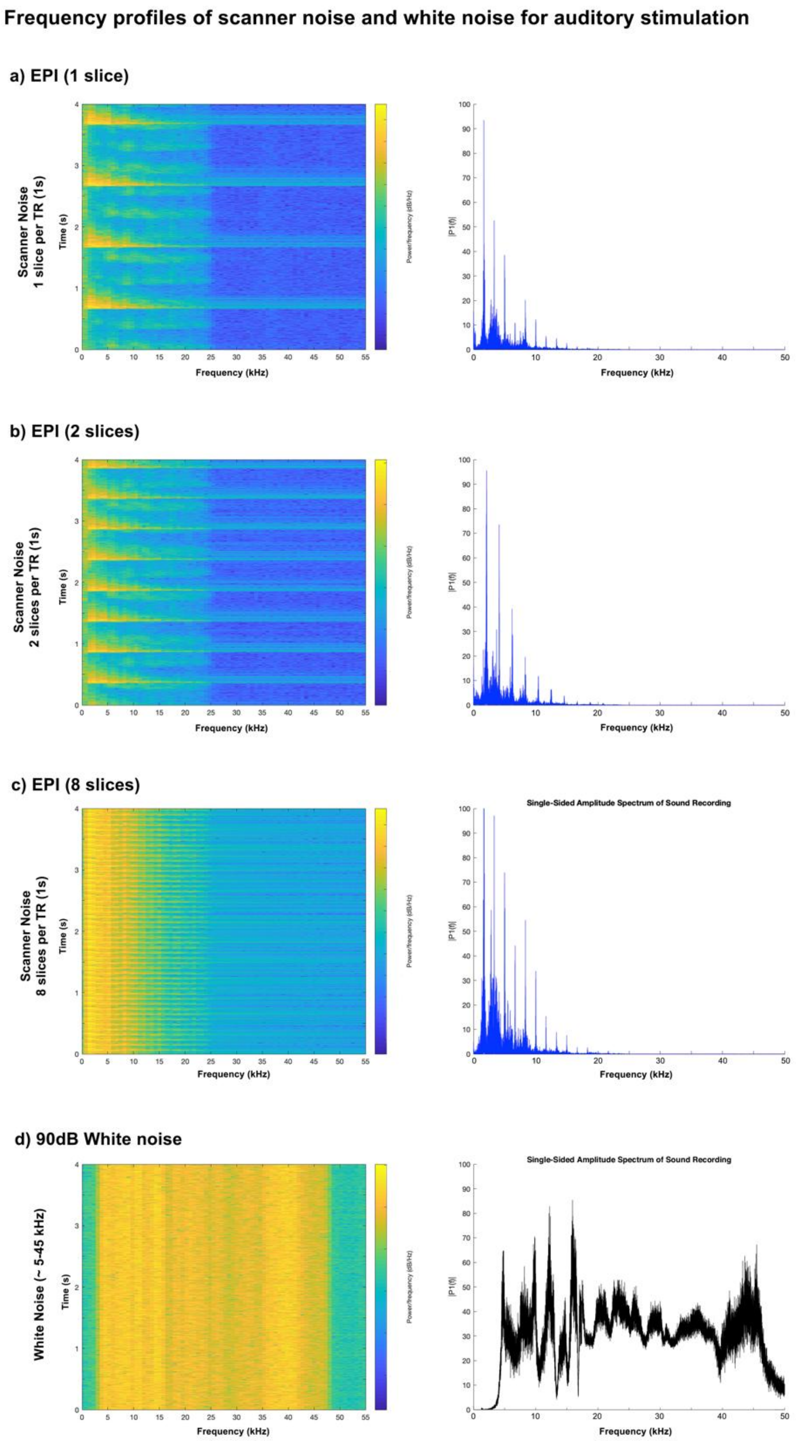

Fig.S2 (A) Frequency profiles of scanner noise with 1 slice acquisition **(B)** 2 slices **(C)** and 8 slices **(D)** 90 dB broadband white noise presented to the animals during the experiments. Total SPL is calculated by summation of the mean square sound pressures of all frequencies. The spectrum was measured 0.5 mm from the distal tip of the sound delivery tube.

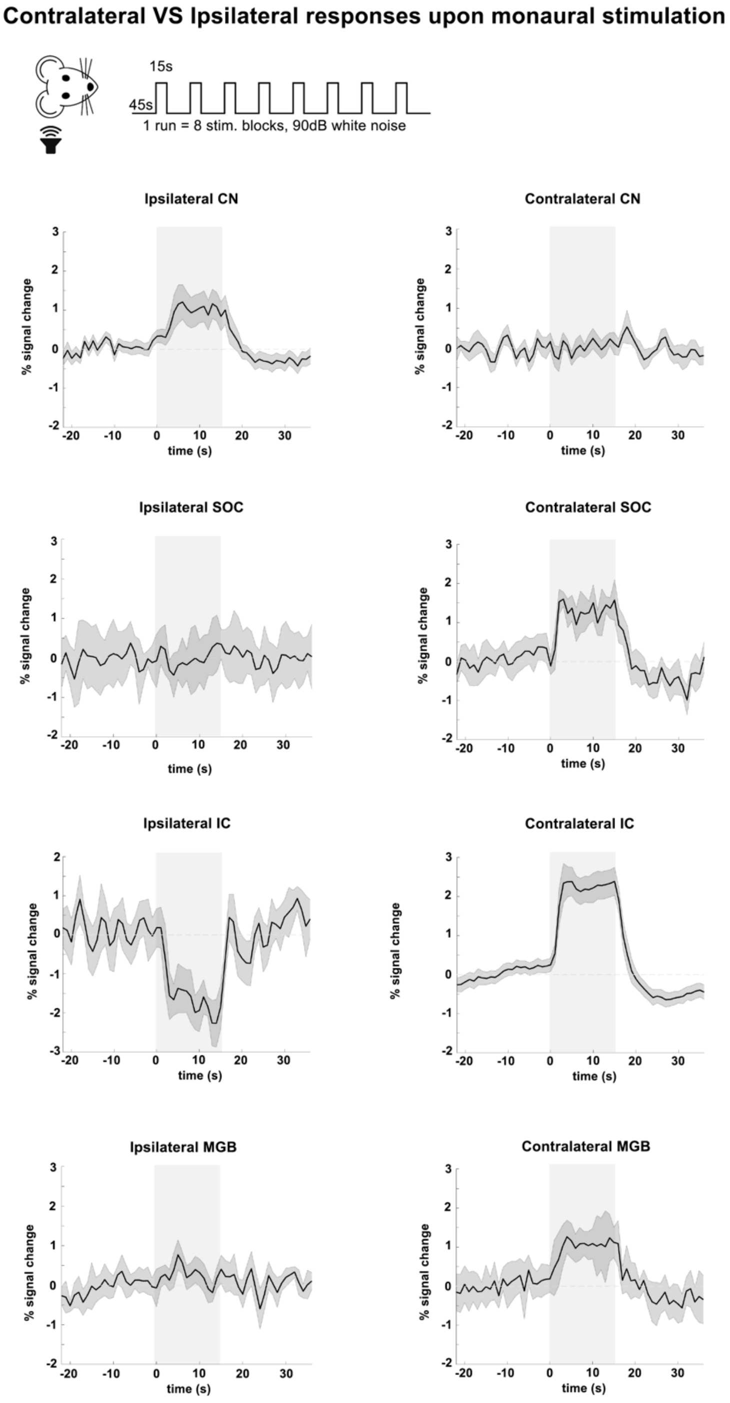

Fig.S3 Contralateral VS Ipsilateral responses upon monaural stimulation. Plots display the time courses of BOLD responses recorded from both the ipsilateral and contralateral sides following monaural auditory stimulation. These responses are shown separately for key auditory processing regions, including the cochlear nucleus (CN), superior olivary complex (SOC), inferior colliculus (IC), and medial geniculate body (MGB).

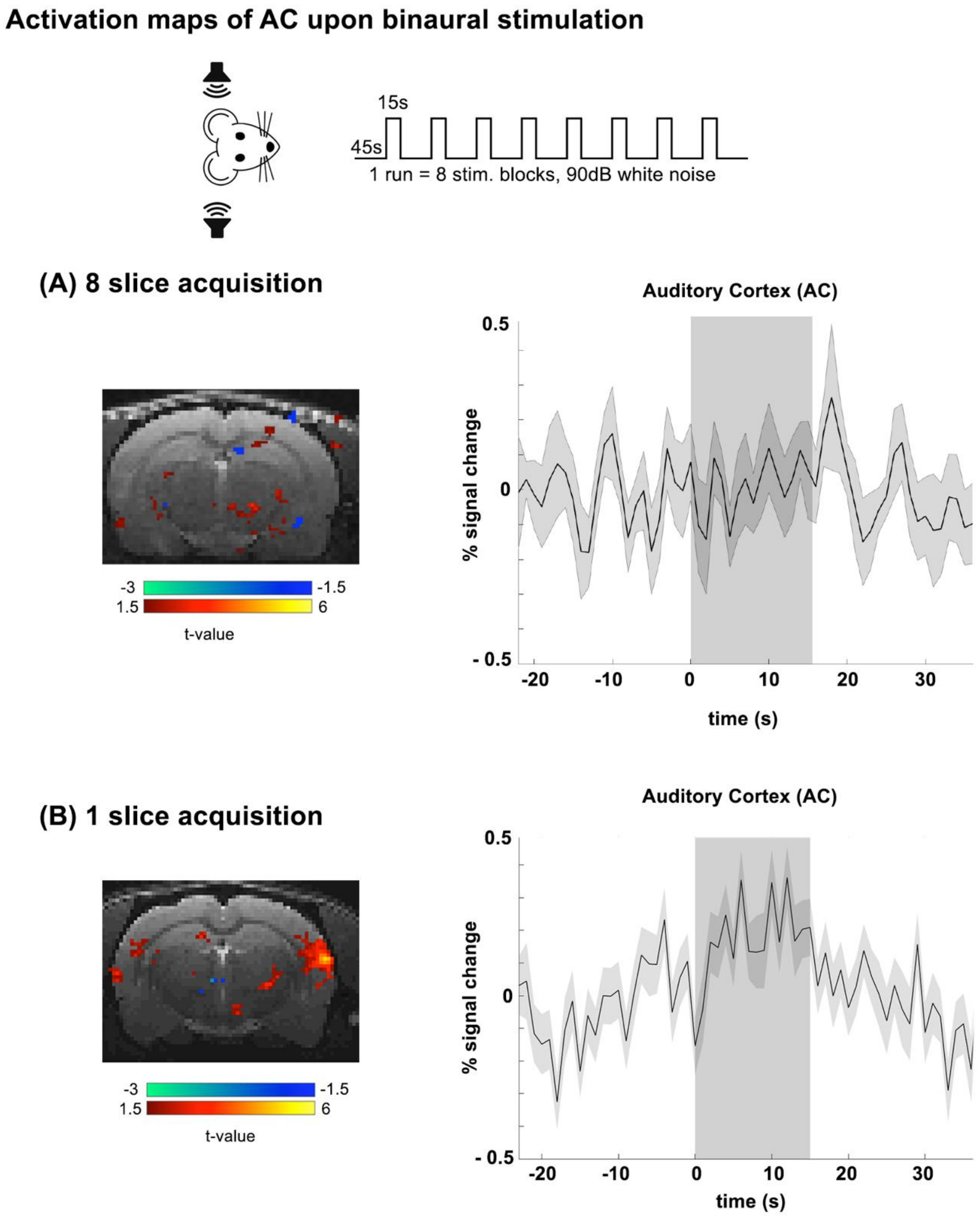

Fig.S4 A) Activation map of the auditory cortex upon binaural stimulation with white noise and averaged time courses for AC upon binaural stimulation during an 8 slice GE-EPI acquisition, showing no AC responses **B)** Activation map of the auditory cortex upon binaural stimulation with white noise and averaged time courses for AC upon binaural stimulation during a 1 slice GE-EPI acquisition, showing minimal AC responses. N = 3.

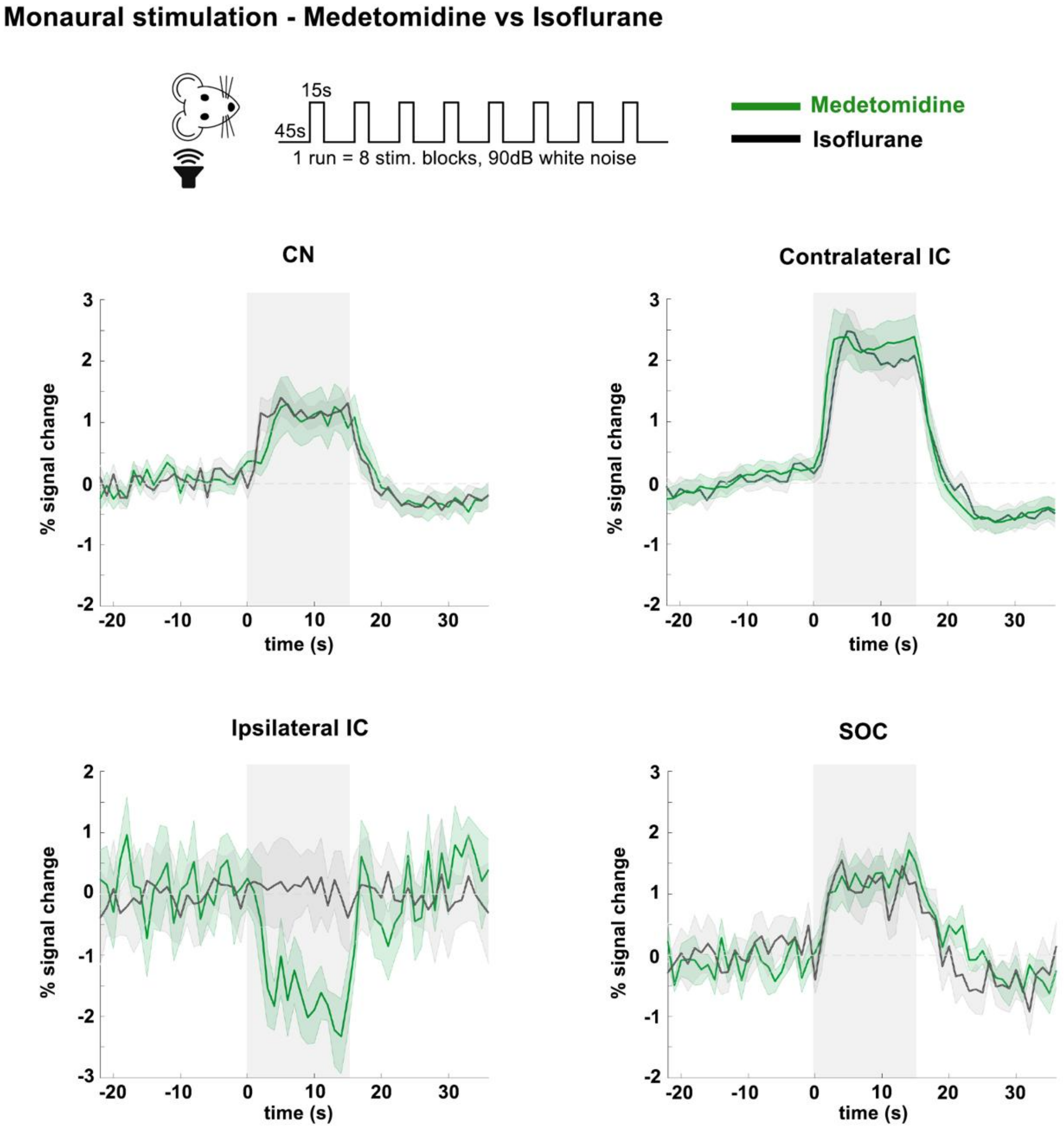

Fig.S5 Comparison between the use of Medetomidine or Isoflurane for auditory fMRI. Plots show BOLD time courses in relevant areas of the auditory pathway upon monaural stimulation. CN, MGB and contralateral IC show no meaningful differences, while the negative BOLD response in the ipsilateral IC is absent in animals scanned under isoflurane. N = 6 (8 slice acquisition).

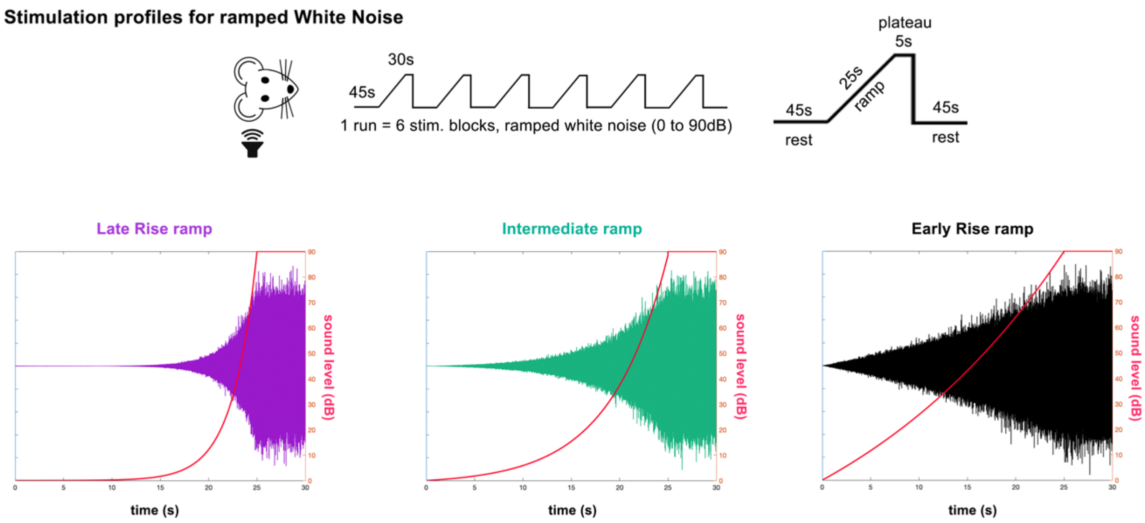

Fig.S6 Ramp profiles of amplitude modulated stimuli. The amplitude “ramped” white noise lasts 25 sec, starting at 0dB and going up to 90dB, followed by a 5 sec 90db plateau and a 45 sec rest, with three distinct envelopes, “Late Rise”, Intermediate” and “Early Rise”.

(A) Sham Lesion - Injection of Saline in Inferior Colicullus (IC)

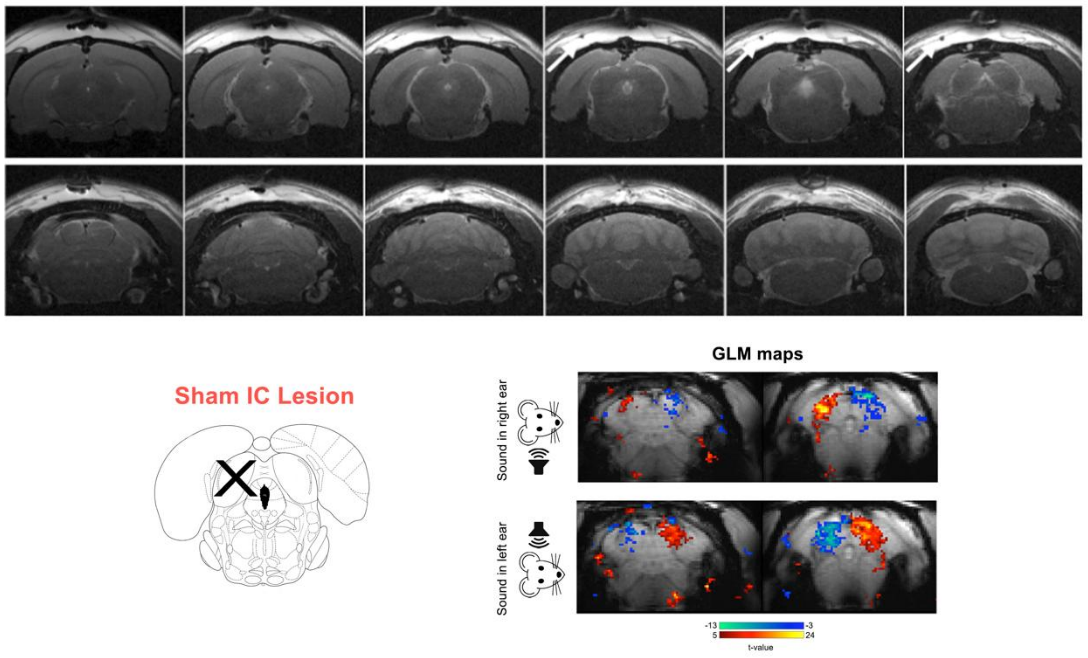

(B) Control Lesion - Injection of Ibotenic Acid in Visual Cortex (V1)

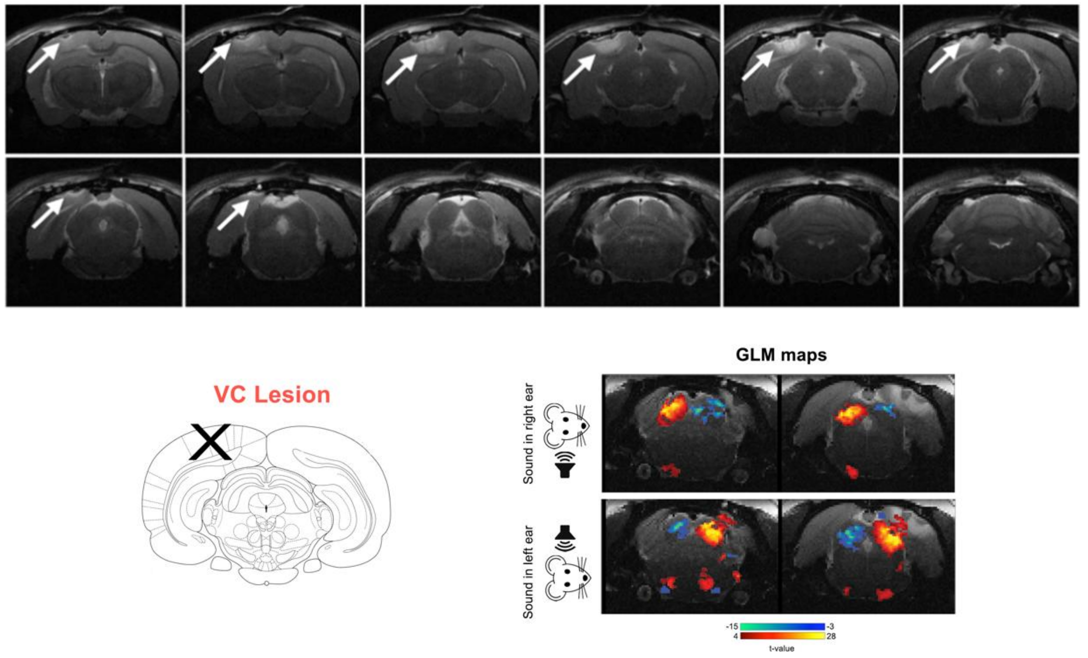

Fig.S7 (A) Sham Lesions with unilateral saline injections in IC at the same volume as ibotenic injections, anatomical and functional maps of monaural responses. "X" denotes the lesioned structure on atlas. N = 2 (2 slice acquisition) **(B)** Visual Cortex lesions with ibotenic acid, anatomical and functional maps of monaural responses. White arrows show the site of injection/lesion. N = 2 (2 slice acquisition).

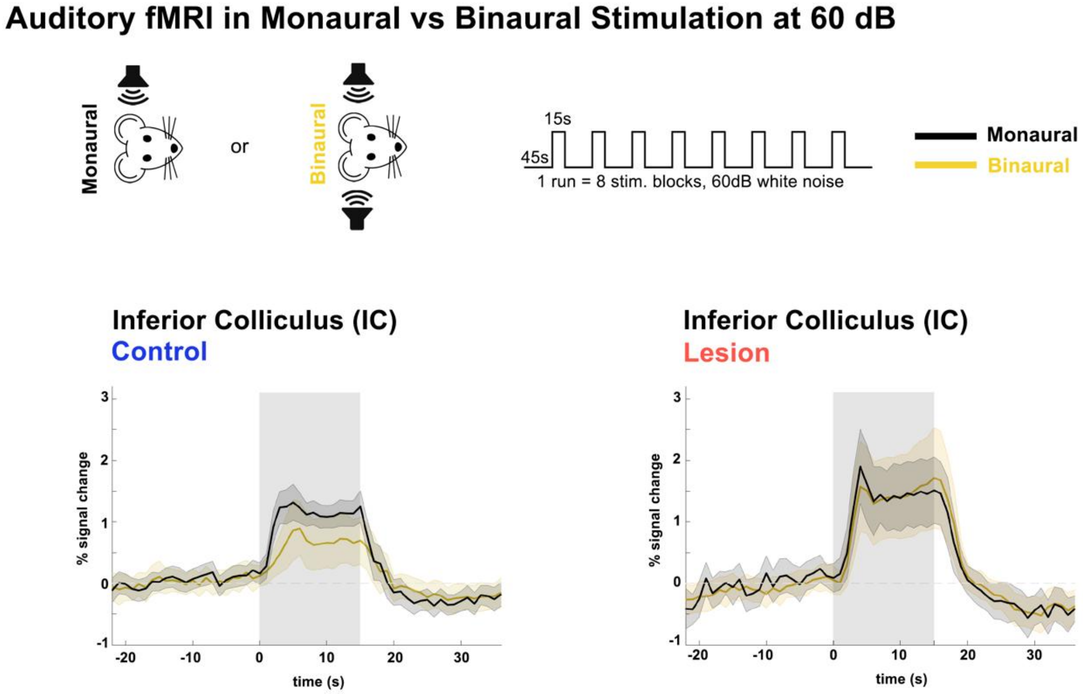

Fig.S8 Auditory fMRI in Monaural vs Binaural Stimulation at 60 dB Plots show time courses of BOLD responses monaural/binaural stimulation in the IC of Control and Lesion groups. Translucid gray bars indicate the stimulation periods. N = 6 (2 slice acquisition).

Supplementary References

Aguilar Ayala, Y., & Malmierca, M. S. (2013). Stimulus-specific adaptation and deviance detection in the inferior colliculus. *Frontiers in Neural Circuits*, *6*. https://doi.org/10.3389/fncir.2012.00089

Aitkin, L. M., & Phillips, S. C. (1984). The interconnections of the inferior colliculi through their commissure. *Journal of Comparative Neurology*, *228*(2), 210–216. https://doi.org/10.1002/cne.902280207

Altman, J. A., Syka, J., & Shmigidina, G. N. (1970). Neuronal activity in the medial geniculate body of the cat during monaural and binaural stimulation. *Experimental Brain Research*, *10*(1), 81–93. https://doi.org/10.1007/BF00340520

Augustinaite, S., & Kuhn, B. (2020). Chronic Cranial Window for Imaging Cortical Activity in Head-Fixed Mice. *STAR Protocols*, *1*(3), 100194. https://doi.org/10.1016/j.xpro.2020.100194

Ayala, Y. A., Pérez-González, D., & Malmierca, M. S. (2016). Stimulus-specific adaptation in the inferior colliculus: The role of excitatory, inhibitory and modulatory inputs. *Biological Psychology*, *116*, 10–22. https://doi.org/10.1016/j.biopsycho.2015.06.016

Bartlett, E. L., Sadagopan, S., & Wang, X. (2011). Fine frequency tuning in monkey auditory cortex and thalamus. *Journal of Neurophysiology*, *106*(2), 849–859. https://doi.org/10.1152/jn.00559.2010

Batra, R., Kuwada, S., & Stanford, T. R. (1993). High-frequency neurons in the inferior colliculus that are sensitive to interaural delays of amplitude-modulated tones: Evidence for dual binaural influences. *Journal of Neurophysiology*, *70*(1), 64–80. https://doi.org/10.1152/jn.1993.70.1.64

Bielefeld, E. C. (2014). Influence of dose and duration of isoflurane anesthesia on the auditory brainstem response in the rat. *International Journal of Audiology*, *53*(4), 250–258. https://doi.org/10.3109/14992027.2013.858280

Blazquez Freches, G., Chavarrias, C., & Shemesh, N. (2018). BOLD-fMRI in the mouse auditory pathway. *NeuroImage*, *165*, 265–277. https://doi.org/10.1016/j.neuroimage.2017.10.027

Brugge, J. F., & Howard, M. A. (2002). Hearing. In V. S. Ramachandran (Ed.), *Encyclopedia of the Human Brain* (pp. 429–448). Academic Press. https://doi.org/10.1016/B0-12-227210-2/00159-X

Brunton, B. W., Botvinick, M. M., & Brody, C. D. (2013). Rats and humans can optimally accumulate evidence for decision-making. *Science (New York, N.Y.)*, *340*(6128), 95–98. https://doi.org/10.1126/science.1233912

Burlingame, R. H., Shrestha, S., Rummel, M. R., & Banks, M. I. (2007). Subhypnotic doses of isoflurane impair auditory discrimination in rats. *Anesthesiology*, *106*(4), 754–762. https://doi.org/10.1097/01.anes.0000264755.24264.68

Caicedo, A., & Herbert, H. (1993). Topography of descending projections from the inferior colliculus to auditory brainstem nuclei in the rat. *The Journal of Comparative Neurology*, *328*(3), 377–392. https://doi.org/10.1002/cne.903280305

Casseday, J. H., Fremouw, T., & Covey, E. (2002). The Inferior Colliculus: A Hub for the Central Auditory System. In D. Oertel, R. R. Fay, & A. N. Popper (Eds.), *Integrative Functions in the Mammalian Auditory Pathway* (pp. 238–318). Springer. https://doi.org/10.1007/978-1-4757-3654-0_7

Chechik, G., Anderson, M. J., Bar-Yosef, O., Young, E. D., Tishby, N., & Nelken, I. (2006). Reduction of information redundancy in the ascending auditory pathway. *Neuron*, *51*(3), 359–368. https://doi.org/10.1016/j.neuron.2006.06.030

Cheung, M. M., Lau, C., Zhou, I. Y., Chan, K. C., Cheng, J. S., Zhang, J. W., Ho, L. C., & Wu, E. X. (2012). BOLD fMRI investigation of the rat auditory pathway and tonotopic organization. *NeuroImage*, *60*(2), 1205–1211. https://doi.org/10.1016/j.neuroimage.2012.01.087

Cook, J. D., Ellinwood, E. H., & Wilson, W. P. (1968). Auditory habituation at primary cortex as a function of stimulus rate. *Experimental Neurology*, *21*(2), 167–175. https://doi.org/10.1016/0014-4886(68)90135-0

Cotillon, N., & Edeline, J. M. (2000). Tone-evoked oscillations in the rat auditory cortex result from interactions between the thalamus and reticular nucleus. *The European Journal of Neuroscience*, *12*(10), 3637–3650. https://doi.org/10.1046/j.1460-9568.2000.00254.x

Davis, K. A. (2002). Evidence of a Functionally Segregated Pathway From Dorsal Cochlear Nucleus to Inferior Colliculus. *Journal of Neurophysiology*, *87*(4), 1824–1835. https://doi.org/10.1152/jn.00769.2001

Dent, M. L., Screven, L. A., & Kobrina, A. (2018). Hearing in Rodents. In M. L. Dent, R. R. Fay, & A. N. Popper (Eds.), *Rodent Bioacoustics* (pp. 71–105). Springer International Publishing. https://doi.org/10.1007/978-3-319-92495-3_4

Devor, A., Tian, P., Nishimura, N., Teng, I. C., Hillman, E. M. C., Narayanan, S. N., Ulbert, I., Boas, D. A., Kleinfeld, D., & Dale, A. M. (2007). Suppressed Neuronal Activity and Concurrent Arteriolar Vasoconstriction May Explain Negative Blood Oxygenation Level-Dependent Signal. *Journal of Neuroscience*, *27*(16), 4452–4459. https://doi.org/10.1523/JNEUROSCI.0134-07.2007

Dubois, J., Berker, A. O. de, & Tsao, D. Y. (2015). Single-Unit Recordings in the Macaque Face Patch System Reveal Limitations of fMRI MVPA. *Journal of Neuroscience*, *35*(6), 2791–2802. https://doi.org/10.1523/JNEUROSCI.4037-14.2015

Eliades, S. J., & Tsunada, J. (2019). Chapter 25—Marmosets in Auditory Research. In R. Marini, L. Wachtman, S. Tardif, K. Mansfield, & J. Fox (Eds.), *The Common Marmoset in Captivity and Biomedical Research* (pp. 451–475). Academic Press. https://doi.org/10.1016/B978-0-12-811829-0.00025-X

Flores, E. N., Duggan, A., Madathany, T., Hogan, A. K., Márquez, F. G., Kumar, G., Seal, R. P., Edwards, R. H., Liberman, M. C., & García-Añoveros, J. (2015). A non-canonical pathway from cochlea to brain signals tissue-damaging noise. *Current Biology: CB*, *25*(5), 606–612. https://doi.org/10.1016/j.cub.2015.01.009

Fox, J. E. (1979). Habituation and prestimulus inhibition of the auditory startle reflex in decerebrate rats. *Physiology & Behavior*, *23*(2), 291–297. https://doi.org/10.1016/0031-9384(79)90370-6

Froemke, R. C., & Jones, B. J. (2011). Development of Auditory Cortical Synaptic Receptive Fields. *Neuroscience and Biobehavioral Reviews*, *35*(10), 2105–2113. https://doi.org/10.1016/j.neubiorev.2011.02.006

Gans, D., Sheykholeslami, K., Peterson, D. C., & Wenstrup, J. (2009). Temporal features of spectral integration in the inferior colliculus: Effects of stimulus duration and rise time. *Journal of Neurophysiology*, *102*(1), 167–180. https://doi.org/10.1152/jn.91300.2008

Geal-Dor, M., Freeman, S., Li, G., & Sohmer, H. (1993). Development of hearing in neonatal rats: Air and bone conducted ABR thresholds. *Hearing Research*, *69*(1–2), 236–242. https://doi.org/10.1016/0378-5955(93)90113-f

Gil, R., Valente, M., Fernandes, F. F., & Shemesh, N. (2024). *Evidence for a push-pull interaction between superior colliculi in monocular dynamic vision mode* (p. 2024.05.06.592678). bioRxiv. https://doi.org/10.1101/2024.05.06.592678

Gil, R., Valente, M., & Shemesh, N. (2024). Rat superior colliculus encodes the transition between static and dynamic vision modes. *Nature Communications*, *15*(1), 849. https://doi.org/10.1038/s41467-024-44934-8

Gil-Loyzaga, P. (2010). Fisiología de receptor y la vía auditiva. In *Fisiología de receptor y la vía auditiva*.

Goense, J. B. M., & Logothetis, N. K. (2008). Neurophysiology of the BOLD fMRI signal in awake monkeys. *Current Biology: CB*, *18*(9), 631–640. https://doi.org/10.1016/j.cub.2008.03.054

Gonzalez-Perez, O., Guerrero-Cazares, H., & Quiñones-Hinojosa, A. (2010). Targeting of Deep Brain Structures with Microinjections for Delivery of Drugs, Viral Vectors, or Cell Transplants. *Journal of Visualized Experiments : JoVE*, *46*, 2082. https://doi.org/10.3791/2082

Greene, N. T., & Davis, K. A. (2019). *Evidence of a Functionally Segregated Pathway from Lateral Superior Olive to Inferior Colliculus* (p. 510354). bioRxiv. https://doi.org/10.1101/510354

Hind, J. E., Goldberg, J. M., Greenwood, D. D., & Rose, J. E. (1963). Some discharge characteristics of single neurons in the inferior colliculus of the cat. II. Timing of the discharges and observations on binaural stimulation. *Journal of Neurophysiology*, *26*, 321–341. https://doi.org/10.1152/jn.1963.26.2.321

Huang, B., Yan, L., Li, Y., Liu, W., Liu, M., Xiao, Z., & Huang, J. (2022). Urethane Improves the Response of Auditory Neurons to Tone. *Frontiers in Cellular Neuroscience*, *16*. https://doi.org/10.3389/fncel.2022.855968

Ito, T. (2020). Different coding strategy of sound information between GABAergic and glutamatergic neurons in the auditory midbrain. *The Journal of Physiology*, *598*(5), 1039–1072. https://doi.org/10.1113/JP279296

Ito, T., Bishop, D. C., & Oliver, D. L. (2016). Functional organization of the local circuit in the inferior colliculus. *Anatomical Science International*, *91*(1), 22–34. https://doi.org/10.1007/s12565-015-0308-8

Ito, T., & Oliver, D. L. (2012). The basic circuit of the IC: Tectothalamic neurons with different patterns of synaptic organization send different messages to the thalamus. *Frontiers in Neural Circuits*, *6*. https://doi.org/10.3389/fncir.2012.00048

Jaramillo, S., & Zador, A. M. (2014). Mice and rats achieve similar levels of performance in an adaptive decision-making task. *Frontiers in Systems Neuroscience*, *8*. https://doi.org/10.3389/fnsys.2014.00173

Kalthoff, D., Po, C., Wiedermann, D., & Hoehn, M. (2013). Reliability and spatial specificity of rat brain sensorimotor functional connectivity networks are superior under sedation compared with general anesthesia. *NMR in Biomedicine*, *26*(6), 638–650. https://doi.org/10.1002/nbm.2908

Kasai, M., Ono, M., & Ohmori, H. (2012). Distinct neural firing mechanisms to tonal stimuli offset in the inferior colliculus of mice *in vivo*. *Neuroscience Research*, *73*(3), 224–237. https://doi.org/10.1016/j.neures.2012.04.009

Kavanagh, G. L., & Kelly, J. B. (1992). Midline and lateral field sound localization in the ferret (Mustela putorius): Contribution of the superior olivary complex. *Journal of Neurophysiology*, *67*(6), 1643–1658. https://doi.org/10.1152/jn.1992.67.6.1643

Kelly, J. B., & Glazier, S. J. (1978). Auditory cortex lesions and discrimination of spatial location by the rat. *Brain Research*, *145*(2), 315–321. https://doi.org/10.1016/0006-8993(78)90865-x

Klingner, C. M., Nenadic, I., Hasler, C., Brodoehl, S., & Witte, O. W. (2011). Habituation within the somatosensory processing hierarchy. *Behavioural Brain Research*, *225*(2), 432–436. https://doi.org/10.1016/j.bbr.2011.07.053

Klug, A., Bauer, E. E., & Pollak, G. D. (1999). Multiple Components of Ipsilaterally Evoked Inhibition in the Inferior Colliculus. *Journal of Neurophysiology*, *82*(2), 593–610. https://doi.org/10.1152/jn.1999.82.2.593

Kuwabara, N., & Zook, J. M. (2000). Geniculo-collicular descending projections in the gerbil. *Brain Research*, *878*(1), 79–87. https://doi.org/10.1016/S0006-8993(00)02695-0

Lau, C., Zhang, J. W., Cheng, J. S., Zhou, I. Y., Cheung, M. M., & Wu, E. X. (2013). Noninvasive fMRI Investigation of Interaural Level Difference Processing in the Rat Auditory Subcortex. *PLOS ONE*, *8*(8), e70706. https://doi.org/10.1371/journal.pone.0070706

Laumen, G., Ferber, A. T., Klump, G. M., & Tollin, D. J. (2016). The Physiological Basis and Clinical Use of the Binaural Interaction Component of the Auditory Brainstem Response. *Ear and Hearing*, *37*(5), e276–e290. https://doi.org/10.1097/AUD.0000000000000301

Lee, H. J., Yoo, S.-J., Lee, S., Song, H.-J., Huh, M.-I., Jin, S.-U., Lee, K.-Y., Lee, J., Cho, J. H., & Chang, Y. (2012). Functional activity mapping of rat auditory pathway after intratympanic manganese administration. *NeuroImage*, *60*(2), 1046–1054. https://doi.org/10.1016/j.neuroimage.2012.01.065

Lee, T.-Y., Weissenberger, Y., King, A. J., & Dahmen, J. C. (2023). Midbrain encodes sound detection behavior without auditory cortex. *eLife*, *12*. https://doi.org/10.7554/eLife.89950.1

Li, L., & Kelly, J. B. (1992). Inhibitory influence of the dorsal nucleus of the lateral lemniscus on binaural responses in the rat's inferior colliculus. *Journal of Neuroscience*, *12*(11), 4530–4539. https://doi.org/10.1523/JNEUROSCI.12-11-04530.1992

Liu, Y., Li, Y., Peng, Y., Yu, H., & Xiao, Z. (2022). Bilateral Interactions in the Mouse Dorsal Inferior Colliculus Enhance the Ipsilateral Neuronal Responses and Binaural Hearing. *Frontiers in Physiology*, *13*. https://doi.org/10.3389/fphys.2022.854077

Lui, L. L., Mokri, Y., Reser, D. H., Rosa, M. G. P., & Rajan, R. (2015). Responses of neurons in the marmoset primary auditory cortex to interaural level differences: Comparison of pure tones and vocalizations. *Frontiers in Neuroscience*, *9*. https://doi.org/10.3389/fnins.2015.00132

Malmierca, M. S. (2006). The Inferior Colliculus: A Center for Convergence of Ascending and Descending Auditory Information. *Neuroembryology and Aging*, *3*(4), 215–229. https://doi.org/10.1159/000096799

Malmierca, M. S. (2015). Chapter 29—Auditory System. In G. Paxinos (Ed.), *The Rat Nervous System (Fourth Edition)* (pp. 865–946). Academic Press. https://doi.org/10.1016/B978-0-12-374245-2.00029-2

Malmierca, M. S., Hernández, O., & Rees, A. (2005). Intercollicular commissural projections modulate neuronal responses in the inferior colliculus. *European Journal of Neuroscience*, *21*(10), 2701–2710. https://doi.org/10.1111/j.1460-9568.2005.04103.x

McQueen, C. (2010). *Comprehensive Toxicology* (2nd Edition). Elsevier Science.

Mei, H., & Chen, Q. (2010). Neural modulation in inferior colliculus and central auditory plasticity. *Frontiers in Biology*, *5*(2), 123–127. https://doi.org/10.1007/s11515-010-0040-7

Mellott, J. G., Foster, N. L., Ohl, A. P., & Schofield, B. R. (2014). Excitatory and inhibitory projections in parallel pathways from the inferior colliculus to the auditory thalamus. *Frontiers in Neuroanatomy*, *8*. https://doi.org/10.3389/fnana.2014.00124

Miller, K., & Covey, E. (2011). Comparison of Auditory Responses in the Medial Geniculate and Pontine Gray of the Big Brown Bat, Eptesicus fuscus. *Hearing Research*, *275*(1–2), 53–65. https://doi.org/10.1016/j.heares.2010.12.001

Murphy, K., Bodurka, J., & Bandettini, P. A. (2007). How long to scan? The relationship between fMRI temporal signal to noise and necessary scan duration. *NeuroImage*, *34*(2), 565–574. https://doi.org/10.1016/j.neuroimage.2006.09.032

Neves, D., Salazar, I. L., Almeida, R. D., & Silva, R. M. (2023). Molecular mechanisms of ischemia and glutamate excitotoxicity. *Life Sciences*, *328*, 121814. https://doi.org/10.1016/j.lfs.2023.121814

Ogawa, S., Lee, T. M., Kay, A. R., & Tank, D. W. (1990). Brain magnetic resonance imaging with contrast dependent on blood oxygenation. *Proceedings of the National Academy of Sciences of the United States of America*, *87*(24), 9868–9872.

Okoyama, S., Moriizumi, T., Kitao, Y., Kawano, J., & Kudo, M. (1995). Anatomical plasticity in the medial superior olive following ablation of the inferior colliculus in neonatal and adult rats. *Hearing Research*, *88*(1), 71–78. https://doi.org/10.1016/0378-5955(95)00101-9

Ono, M., & Ito, T. (2018). Inhibitory Neural Circuits in the Mammalian Auditory Midbrain. *Journal of Experimental Neuroscience*, *12*, 1179069518818230. https://doi.org/10.1177/1179069518818230

Ortiz-Rios, M., Azevedo, F. A. C., Kuśmierek, P., Balla, D. Z., Munk, M. H., Keliris, G. A., Logothetis, N. K., & Rauschecker, J. P. (2017). Widespread and Opponent fMRI Signals Represent Sound Location in Macaque Auditory Cortex. *Neuron*, *93*(4), 971-983.e4. https://doi.org/10.1016/j.neuron.2017.01.013

Orton, L. D., Papasavvas, C. A., & Rees, A. (2016). Commissural Gain Control Enhances the Midbrain Representation of Sound Location. *Journal of Neuroscience*, *36*(16), 4470–4481. https://doi.org/10.1523/JNEUROSCI.3012-15.2016

Orton, L. D., & Rees, A. (2014). Intercollicular commissural connections refine the representation of sound frequency and level in the auditory midbrain. *eLife*, *3*, e03764. https://doi.org/10.7554/eLife.03764

Osanai, H., & Tateno, T. (2016). Neural response differences in the rat primary auditory cortex under anesthesia with ketamine versus the mixture of medetomidine, midazolam and butorphanol. *Hearing Research*, *339*, 69–79. https://doi.org/10.1016/j.heares.2016.06.012

O'Shaughnessy, B. (1957). The Location of Sound. *Mind*, *66*(264), 471–490.

Paasonen, J., Stenroos, P., Salo, R. A., Kiviniemi, V., & Gröhn, O. (2018). Functional connectivity under six anesthesia protocols and the awake condition in rat brain. *NeuroImage*, *172*, 9–20. https://doi.org/10.1016/j.neuroimage.2018.01.014

Pai, S., Erlich, J. C., Kopec, C., & Brody, C. D. (2011). Minimal Impairment in a Rat Model of Duration Discrimination Following Excitotoxic Lesions of Primary Auditory and Prefrontal Cortices. *Frontiers in Systems Neuroscience*, *5*, 74. https://doi.org/10.3389/fnsys.2011.00074

Pardo-Vazquez, J. L., Castiñeiras-de Saa, J. R., Valente, M., Damião, I., Costa, T., Vicente, M. I., Mendonça, A. G., Mainen, Z. F., & Renart, A. (2019). The mechanistic foundation of Weber's law. *Nature Neuroscience*, *22*(9), 1493–1502. https://doi.org/10.1038/s41593-019-0439-7

Paxinos, G., & Watson, C. (2009). *The Rat Brain in Stereotaxic Coordinates*. Elsevier/Academic.

Ping, J., Li, N., Galbraith, G. C., Wu, X., & Li, L. (2008). Auditory frequency-following responses in rat ipsilateral inferior colliculus. *Neuroreport*, *19*(14), 1377–1380. https://doi.org/10.1097/WNR.0b013e32830c1cfa

Poellinger, A., Thomas, R., Lio, P., Lee, A., Makris, N., Rosen, B. R., & Kwong, K. K. (2001). Activation and Habituation in Olfaction—An fMRI Study. *NeuroImage*, *13*(4), 547–560. https://doi.org/10.1006/nimg.2000.0713

Poeppel, D., Overath, T., Popper, A. N., & Fay, R. R. (Eds.). (2012). *The Human Auditory Cortex* (Vol. 43). Springer. https://doi.org/10.1007/978-1-4614-2314-0

Pollak, G. D. (2012). Circuits for processing dynamic interaural intensity disparities in the inferior colliculus. *Hearing Research*, *288*(1), 47–57. https://doi.org/10.1016/j.heares.2012.01.011

Prado-Gutierrez, P., Castro-Fariñas, A., Morgado-Rodriguez, L., Velarde-Reyes, E., Martínez, A. D., & Martínez-Montes, E. (2015). Habituation of Auditory Steady State Responses Evoked by Amplitude-Modulated Acoustic Signals in Rats. *Audiology Research*, *5*(1), 113. https://doi.org/10.4081/audiores.2015.113

Rabe, K., Michael, N., Kugel, H., Heindel, W., & Pfleiderer, B. (2006). fMRI studies of sensitivity and habituation effects within the auditory cortex at 1.5 T and 3 T. *Journal of Magnetic Resonance Imaging*, *23*(4), 454–458. https://doi.org/10.1002/jmri.20547

Raghavendra, M., Maiti, R., Kumar, S., & Acharya, S. (2013). Role of aqueous extract of Azadirachta indica leaves in an experimental model of Alzheimer's disease in rats. *International Journal of Applied & Basic Medical Research*, *3*(1), 37–47. https://doi.org/10.4103/2229-516X.112239

Rosburg, T., Trautner, P., Boutros, N. N., Korzyukov, O. A., Schaller, C., Elger, C. E., & Kurthen, M. (2006). Habituation of auditory evoked potentials in intracranial and extracranial recordings. *Psychophysiology*, *43*(2), 137–144. https://doi.org/10.1111/j.1469-8986.2006.00391.x

Saldaña, E., & Merchán, M. A. (1992). Intrinsic and commissural connections of the rat inferior colliculus. *The Journal of Comparative Neurology*, *319*(3), 417–437. https://doi.org/10.1002/cne.903190308

Sally, S. L., & Kelly, J. B. (1992). Effects of superior olivary complex lesions on binaural responses in rat inferior colliculus. *Brain Research*, *572*(1–2), 5–18. https://doi.org/10.1016/0006-8993(92)90444-e

Samson, F. K., Barone, P., Irons, W. A., Clarey, J. C., Poirier, P., & Imig, T. J. (2000). Directionality Derived From Differential Sensitivity to Monaural and Binaural Cues in the Cat's Medial Geniculate Body. *Journal of Neurophysiology*, *84*(3), 1330–1345. https://doi.org/10.1152/jn.2000.84.3.1330

Schwinn, D. A., McIntyre, R. W., & Reves, J. G. (1990). Isoflurane-Induced Vasodilation: Role of the α-Adrenergic Nervous System. *Anesthesia & Analgesia*, *71*(5), 451.

Semple, M. N., & Kitzes, L. M. (1985). Single-unit responses in the inferior colliculus: Different consequences of contralateral and ipsilateral auditory stimulation. *Journal of Neurophysiology*, *53*(6), 1467–1482. https://doi.org/10.1152/jn.1985.53.6.1467

Sheppard, A. M., Chen, G.-D., Manohar, S., Ding, D., Hu, B.-H., Sun, W., Zhao, J., & Salvi, R. (2017). Prolonged low-level noise-induced plasticity in the peripheral and central auditory system of rats. *Neuroscience*, *359*, 159–171. https://doi.org/10.1016/j.neuroscience.2017.07.005

Shmuel, A., Augath, M., Oeltermann, A., & Logothetis, N. K. (2006). Negative functional MRI response correlates with decreases in neuronal activity in monkey visual area V1. *Nature Neuroscience*, *9*(4), 569–577. https://doi.org/10.1038/nn1675

Siero, J. C. W., Hermes, D., Hoogduin, H., Luijten, P. R., Ramsey, N. F., & Petridou, N. (2014). BOLD matches neuronal activity at the mm scale: A combined 7 T fMRI and ECoG study in human sensorimotor cortex. *NeuroImage*, *101*, 177–184. https://doi.org/10.1016/j.neuroimage.2014.07.002

Soga, R., Shiramatsu, T. I., & Takahashi, H. (2018). Preference test of sound among multiple alternatives in rats. *PLOS ONE*, *13*(6), e0197361. https://doi.org/10.1371/journal.pone.0197361

Solyga, M., & Barkat, T. R. (2021). Emergence and function of cortical offset responses in sound termination detection. *eLife*, *10*, e72240. https://doi.org/10.7554/eLife.72240

Souffi, S., Nodal, F. R., Bajo, V. M., & Edeline, J.-M. (2021). When and How Does the Auditory Cortex Influence Subcortical Auditory Structures? New Insights About the Roles of Descending Cortical Projections. *Frontiers in Neuroscience*, *15*. https://doi.org/10.3389/fnins.2021.690223

Steinschneider, M., Nourski, K. V., & Fishman, Y. I. (2013). Representation of speech in human auditory cortex: Is it special? *Hearing Research*, *305*, 57–73. https://doi.org/10.1016/j.heares.2013.05.013

Sten, S., Lundengård, K., Witt, S. T., Cedersund, G., Elinder, F., & Engström, M. (2017). Neural inhibition can explain negative BOLD responses: A mechanistic modelling and fMRI study. *NeuroImage*, *158*, 219–231. https://doi.org/10.1016/j.neuroimage.2017.07.002

van Alst, T. M., Wachsmuth, L., Datunashvili, M., Albers, F., Just, N., Budde, T., & Faber, C. (2019). Anesthesia differentially modulates neuronal and vascular contributions to the BOLD signal. *NeuroImage*, *195*, 89–103. https://doi.org/10.1016/j.neuroimage.2019.03.057

Wade, A. R. (2002). The Negative BOLD Signal Unmasked. *Neuron*, *36*(6), 993–995. https://doi.org/10.1016/S0896-6273(02)01138-8

Wei, J., Zhong, W., Xiao, C., Liu, Y., Song, C., & Xiao, Z. (2018). Selectivity of Monaural Synaptic Inputs Underlying Binaural Auditory Information Integration in the Central Nucleus of Inferior Colliculus. *Frontiers in Cellular Neuroscience*, *12*. https://doi.org/10.3389/fncel.2018.00303

Westenberg, I. S., & Weinberger, N. M. (1976). Evoked potential decrements in auditory cortex. II. Critical test for habituation. *Electroencephalography and Clinical Neurophysiology*, *40*(4), 356–369. https://doi.org/10.1016/0013-4694(76)90187-5

Wilson, D. A. (1998). Synaptic Correlates of Odor Habituation in the Rat Anterior Piriform Cortex. *Journal of Neurophysiology*, *80*(2), 998–1001. https://doi.org/10.1152/jn.1998.80.2.998

Xie, H., Chung, D. Y., Kura, S., Sugimoto, K., Aykan, S. A., Wu, Y., Sakadžić, S., Yaseen, M. A., Boas, D. A., & Ayata, C. (2020). Differential effects of anesthetics on resting state functional connectivity in the mouse. *Journal of Cerebral Blood Flow & Metabolism*, *40*(4), 875–884. https://doi.org/10.1177/0271678X19847123

Xiong, X. R., Liang, F., Li, H., Mesik, L., Zhang, K. K., Polley, D. B., Tao, H. W., Xiao, Z., & Zhang, L. I. (2013). Interaural level difference-dependent gain control and synaptic scaling underlying binaural computation. *Neuron*, *79*(4), 738–753. https://doi.org/10.1016/j.neuron.2013.06.012

Xu, N., LaGrow, T. J., Anumba, N., Lee, A., Zhang, X., Yousefi, B., Bassil, Y., Clavijo, G. P., Khalilzad Sharghi, V., Maltbie, E., Meyer-Baese, L., Nezafati, M., Pan, W.-J., & Keilholz, S. (2022). Functional Connectivity of the Brain Across Rodents and Humans. *Frontiers in Neuroscience*, *16*. https://doi.org/10.3389/fnins.2022.816331

Zhang, J. W., Lau, C., Cheng, J. S., Xing, K. K., Zhou, I. Y., Cheung, M. M., & Wu, E. X. (2013). Functional magnetic resonance imaging of sound pressure level encoding in the rat central auditory system. *NeuroImage*, *65*, 119–126. https://doi.org/10.1016/j.neuroimage.2012.09.069

Zhao, L., Liu, Y., Shen, L., Feng, L., & Hong, B. (2011). Stimulus-specific adaptation and its dynamics in the inferior colliculus of rat. *Neuroscience*, *181*, 163–174. https://doi.org/10.1016/j.neuroscience.2011.01.060
